# Quantifying intra‐ and inter‐species contact rates at supplemental feeding sites in Ethiopia to inform rabies maintenance potential of multiple host species

**DOI:** 10.1111/tbed.14755

**Published:** 2022-11-15

**Authors:** Laura Binkley, Jeanette O'Quin, Balbine Jourdan, Getnet Yimer, Asefa Deressa, Laura W. Pomeroy

**Affiliations:** ^1^ Department of Veterinary Preventive Medicine College of Veterinary Medicine The Ohio State University Columbus Ohio USA; ^2^ Global One Health initiative Office of Internaional Affairs, The Ohio State University Columbus Ohio USA; ^3^ College of Veterinary Medicine The Ohio State University Columbus Ohio USA; ^4^ Rabies and Other Zoonotic Diseases Research Division Ethiopian Public Health Institute Addis Ababa Ethiopia; ^5^ Environmental Health Sciences College of Public Health The Ohio State University Columbus Ohio USA; ^6^ Translational Data Analytics Institute The Ohio State University Columbus Ohio USA

**Keywords:** basic reproductive number (*R*_0_), contact rate, cross‐species, maintenance, rabies, reservoir, wildlife, zoonotic

## Abstract

Rabies, a multi‐host pathogen responsible for the loss of roughly 59,000 human lives each year worldwide, continues to impose a significant burden of disease despite control efforts, especially in Ethiopia. However, how species other than dogs contribute to rabies transmission throughout Ethiopia remains largely unknown. In this study, we quantified interactions among wildlife species in Ethiopia with the greatest potential for contributing to rabies maintenance. We observed wildlife at supplemental scavenging sites across multiple landscape types and quantified transmission potential. More specifically, we used camera trap data to quantify species abundance, species distribution, and intra‐ and inter‐species contacts per trapping night over time and by location. We derived a mathematical expression for the basic reproductive number (*R*_0_) based on within‐ and between‐species contract rates by applying the next generation method to the susceptible, exposed, infectious, removed model. We calculated *R*_0_ for transmission within each species and between each pair of species using camera trap data in order to identify pairwise interactions that contributed the most to transmission in an ecological community. We estimated which species, or species pairs, could maintain transmission ($R_{0}>1$) and which species, or species pairs, had contact rates too low for maintenance ($R_{0}<1$). Our results identified multiple urban carnivores as candidate species for rabies maintenance throughout Ethiopia, with hyenas exhibiting the greatest risk for rabies maintenance through intra‐species transmission. Hyenas and cats had the greatest risk for rabies maintenance through inter‐species transmission. Urban and peri‐urban sites posed the greatest risk for rabies transmission. The night‐time hours presented the greatest risk for a contact event that could result in rabies transmission. Overall, both intra‐ and inter‐species contacts posed risk for rabies maintenance. Our results can be used to target future studies and inform population management decisions.

## 1 INTRODUCTION

Rabies has been a critical health threat worldwide for centuries. Currently, roughly 59,000 people die from rabies worldwide every year, despite the disease being completely preventable (Hampson et al., 2015). Vaccination has eliminated canine rabies in some parts of the world where dogs maintain the virus, but other locations still experience a significant disease burden maintained by a multi‐species host reservoir. Made up of one or more epidemiologically connected populations, a reservoir can maintain pathogens and periodically transmit them to external target populations by spillover (Haydon et al., 2002; Viana et al., 2014). In many parts of the world where rabies persists, it is difficult to identify which species can maintain transmission independently, which species maintain transmission as part of a reservoir maintenance community, and which species became infected during a spillover event. Fine‐scale data on all wildlife species within a geographic area is needed to identify the role that each plays in rabies maintenance. For multi‐host pathogens, it is essential to have a clear understanding of existing reservoir populations in order to properly target control and elimination efforts.

One approach to identify which wildlife species comprise the reservoir for a multi‐host pathogen, such as rabies, is to determine which species are connected by epidemiologically relevant contact both within and between species (Fenton & Pedersen, 2005; Viana et al., 2014). This type of fine‐scale epidemiological information can be difficult to obtain from wildlife populations, especially in the absence of an outbreak, because the identification of disease state often requires capturing and testing of large numbers of animals through invasive methods. However, we can observe contacts in healthy animals and apply these data, along with other known epidemiological parameters, to mathematical functions that inform transmission dynamics (Anderson et al., 1981). Rabies transmission can be quantified as the rate at which any individuals in the population contact each other (*c*), the proportion (*p*) of the population that is infectious, and the combined probability (*v*) of a bite with the probability of transmission over that bite (Begon et al., 2002). This transmission quantity, also known as the force of infection, impacts *R*_0_, or the average number of secondary infections caused by a primary infection when introduced to a completely susceptible host population, also known as the basic reproductive ratio. When $R_{0}\geq 1$, each primary case causes one or more secondary cases, and transmission is maintained. When $R_{0}<1$, each primary case does not cause a secondary case, and transmission is not maintained. The value for the number of contacts, the force of infection and *R*_0_ can imply transmission dynamics ranging from dead‐end cases to stuttering chains of transmission or large outbreaks that can sustain to the point of endemicity (Viana et al., 2014).

Multiple tools have been used to identify contacts in wildlife populations. Radiotelemetry, global positioning systems (GPS) and passive integrated transponder tags have previously been used to quantify contact rates in wild animal populations (Real & Biek, 2007; Ryder et al., 2012). However, these methods are costly, time intensive and often underestimate the number of contacts unless all individuals are tagged or the proportion of the population that is tagged is known (Real & Biek, 2007). Moreover, these methods can also be especially challenging to use with large carnivores that are difficult to trap and can easily remove such devices. Recent advances in camera trap technology, such as inclusion of infrared night vision recording capacity, have increased our ability to monitor populations at levels that can provide this valuable quantitative data without the need to capture animals. Camera traps are low‐labour, low‐cost, non‐invasive, robust to variation in ground conditions and climate, can be used to gain information from highly cryptic species, and are equally efficient at collecting data by day and night (Rowcliffe et al., 2008; Trolliet et al., 2014). They also allow simultaneous data collection on multiple species in addition to providing the ability to observe wild animals, undisturbed, in their natural environment. Although still considered a form of indirect observation, camera traps allow for the observation of animal behaviours and further, images can then be linked with other data such as date, time and temperature. Strategic placement of camera traps at resource caches, such as supplemental scavenging sites, can provide data on interactions, including both intra‐ and inter‐species contacts. These data can then be used to calculate contact rates at different points throughout the day or night and at different locations (Boitani & Powell, 2012; Kelly et al., 2012).

Ethiopia serves as a model system for implementing such methods to examine rabies transmission dynamics in wild animal populations. Rabies remains a priority disease in Ethiopia (Pieracci et al., 2016) with an estimated 2700 humans dying of rabies in Ethiopia every year, making it the country with the second highest annual number of human rabies deaths in Africa (Coetzer et al., 2016; Hampson et al., 2015). Most major wildlife reservoir hosts of rabies (e.g. mongoose, jackal, fox) are opportunistic species that live at relatively high densities in agricultural areas or close to human settlements and frequently interact with dogs (Cleaveland & Dye, 1995; Craft et al., 2017). Ethiopia is a country with nearly 115 million people (World Bank, 2022) where agriculture is the main source of income (United Nations, 2017), biodiversity is declining (Critical Ecosystem Partnership Fund, 2017), and stable populations of opportunistic species reside. This environment provides an abundance of grazing areas, water points and backyard or slaughterhouse waste disposal areas that act as supplemental scavenging sites. These areas attract large numbers of individuals from different species to the same location, thus serving as areas of high risk for pathogen transmission both within and between species and can facilitate the circulation of the virus throughout the country (Deressa et al., 2010). Consequently, the frequency of species interactions increases in these areas where the public report frequent predation on dogs and livestock by wildlife species (Mojo et al., 2014). However, contact rates within and between animal populations at such high‐risk areas in Ethiopia have never been quantified.

Contact between species is not the only criteria for cross‐species transmission of rabies. Viral characteristics, innate immune response, host immunology and physiological factors associated with both host and pathogen genetic relatedness are also important factors (Plowright et al., 2017; Fisher et al. 2018). Many rabies variants are host‐adapted and maintained in distinct host‐associated transmission cycles (Mollentze et al., 2014), resulting in clades or biotypes relating to the local fauna (Hayman et al., 2016). Nevertheless, there is evidence for cross‐species transmission of host‐adapted rabies strains (Wallace et al., 2014). Dog‐adapted rabies variants are more likely to transmit to other species than rabies variants adapted to other species (Bonnaud et al., 2019), suggesting that the predominant dog rabies variant of the Africa 1a clade circulating in Ethiopia (Deressa et al., 2015; Johnson et al., 2010; Marston et al., 2015; Zulu et al., 2009), might transmit across species, especially to the order Carnivora. Phylogenetic analysis of Ethiopian rabies viral isolates suggests repeated introductions from dogs to the endangered Ethiopian wolf population (Randall et al., 2004), from dogs to jackals (Binkley et al., 2021) and from dogs to donkeys (Binkley et al., 2021). Species belonging to the Mustelidae (ferret, badger), Herpestidae (mongoose) and Mephitidae (skunks) families are also in the order carnivora and remain candidates for cross‐species transmission from dogs (Troupin et al., 2016).

The spotted hyena (*Crocuta crocuta*), referred to as hyena in this paper, and African golden wolf (*Canis anthus*), previously known as common jackal and referred to as wolf in this paper, are carnivores that have not only been some of the most frequently reported livestock raiding species in Ethiopia, but sightings of these species have also been reported frequently during peaks in rabies cases in different parts of the country (Okell et al., 2013). The hyena is of special interest because they exist at very high densities in certain parts of the country in addition to being able to survive almost entirely off of anthropogenic food sources (Yirga et al., 2013). As a result, they have lost their clan structure in certain parts of the country, allowing them to exist as large groups with little social structure (Schramme, 2015). Hyenas in Ethiopia are also known to move in and out of large cities and towns throughout the night, often coming into close contact with dogs guarding households and potentially serving as vehicles for rabies transmission from rural to urban areas and vice versa (Mebatsion et al., 1992). These species, along with other carnivores reported to carry rabies in Ethiopia such as various fox, jackal, wolf and mongoose species, serve as candidate species for independent rabies maintenance or to serve as part of multi‐species host reservoir (Deressa et al., 2013; Johnson et al., 2010).

The current study uses camera trap data collected from supplemental scavenging sites throughout Ethiopia to characterize and quantify both intra‐ and inter‐species contact rates and explore which wildlife species contribute to rabies dynamics in different parts of the country. Specifically, we will (1) compare species abundance and contact rates across landscape types; (2) compare species abundance and contact rates over 17‐h recording periods; (3) identify multi‐species contact networks; and (4) identify which species have the greatest probability of being able to maintain rabies transmission, either as a single species or within a species pair, solely based on ecological opportunity and contact structure. This information is necessary to identify which species interactions are the most high risk for pathogen transmission.

## 2 METHODS

### 2.1 Site selection

**FIGURE 1:**
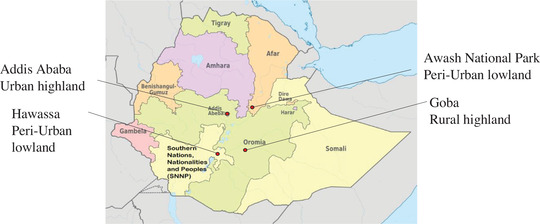
**Camera trap survey locations by landscape classification** (Ethiopian Central Statistics Agency, 2013). Camera trap surveys were conducted in Addis Ababa, an urban highland site, in Hawassa, a peri‐urban lowland site, in Awash, a peri‐urban lowland site, and in Goba, a rural highland site.

We selected study areas to incorporate urban and rural landscapes as well as highland and lowland habitats. Our study sites were located in different regions of Ethiopia. The Addis Ababa and Goba sites are located in the Oromia region, the Awash site is located in the Afar region, and the Hawassa site is located in the Southern Nations Nationalities and Peoples’ region (Figure 1; Table S1). To select sites for camera traps within each municipality, we visited each central municipality office to help us identify a primary slaughter plant facility that practised uncontained scrap disposal, thus allowing for open feeding.

### 2.2 Camera trap setup

We utilized four Reconyx Hyperfire model PC900 camera traps specialized for covert wildlife research (Reconyx, Holmen, WI). These models captured high‐quality images (1080HD day/monochrome at night), recorded at night (no glow infrared night vision), stored large amounts of information (32GB), resisted harsh weather conditions and performed at a range of temperatures (between −40°C and 60°C).

Our cameras were attached to trees at 1–2 m above the ground and to pole mounts at roughly 0.75 m above ground when trees were not available. Height above ground was selected to be slightly above eye level for most canid species. We mounted all cameras at a slightly downward angle of roughly 5° below horizontal as recommended by the manufacturer, which produced a field of view of 35°. We checked cameras every other day and restored cameras to their original position if they had been moved.

We used the time lapse setting and took one photograph per minute over 17‐h periods starting at 6:00 PM (1800) and ending at 11:00 AM (1100), allowing observation at times of peak activity for nocturnal, diurnal and crepuscular species. This period will be referred to as a trapping night throughout the remainder of this text. We initially used both the time lapse setting in combination with the trigger setting; however, memory storage filled too quickly, and more photos were taken than minutes recorded. The full trapping night could not be captured on several occasions due to human interference (e.g. obstruction or stealing of the camera). We recorded over a minimum of three trapping nights at each site to remain consistent with the estimated infectious period for rabies (3.1 days) (Hampson et al., 2009), ranging from 3 to 13 trapping nights at each site (Table S2).

### 2.3 Species abundance and contact

**FIGURE 2:**
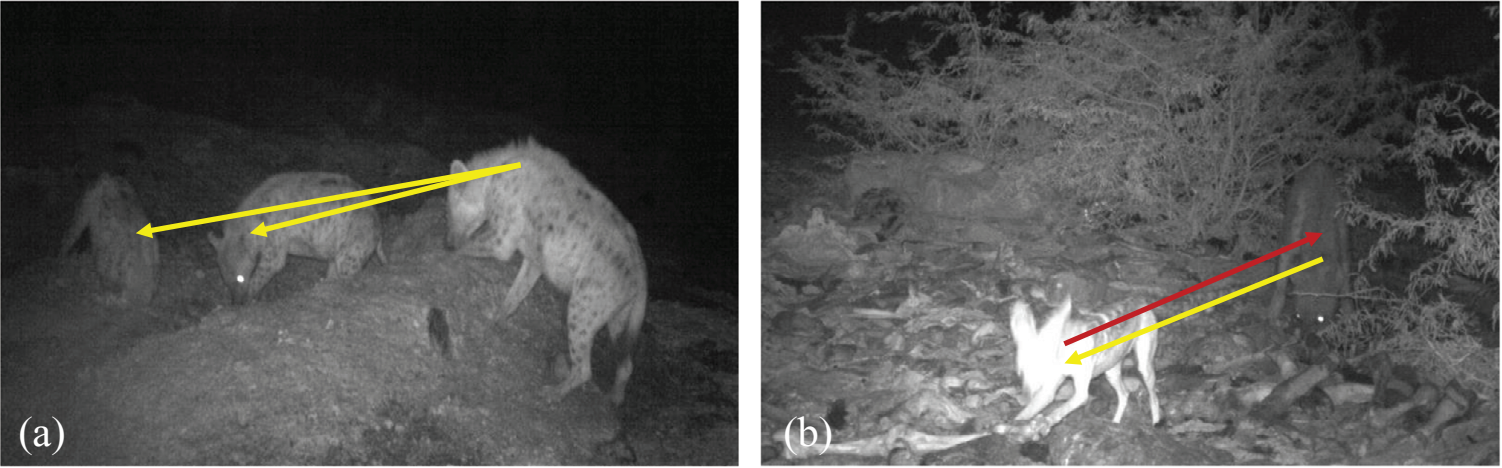
**Intra‐ and inter‐species contacts**. (a) Arrows indicate intra‐species contacts or network edges. A single rabid individual would have the opportunity to bite two other individuals of the same species. (b) Arrows indicate inter‐species contacts or network edges. The yellow arrow indicates hyena‐to‐jackal transmission and the red arrow indicates jackal‐to‐hyena transmission. Taken together, these two arrows indicate that potential for bidirectional transmission. Each species has an opportunity for one inter‐species contact.

We manually recorded the study site, time, date and number of individuals present by species in each photo. We aimed to defined intra‐species contacts as the number of individuals of the same species *i* that any single individual at site *l* encountered over a trapping night *k* ($c_{i,i,l,k}$). Due to the inability of current technology to easily and accurately distinguish individuals in camera trap photos, especially during night‐time recording, we used maximum numbers of individuals within a single photo per trapping night as a proxy to ensure that contacts were quantified as independent events among unique individuals as opposed to repeat measures (Figure 2a). We assumed proximity as a prerequisite for bites and accounted for a reduced probability of bites compared to proximity in later steps (see Table 1). We also assumed that any individual could contact all other individuals of the same species within each photo. We quantified contacts by, first, counting all individuals of species *i* ($n_{i}$) captured within each single photo and grouping by site (*l*) and night (*k*) into the vector $n_{i,l,k}$. Second, we calculated the number of intra‐species contacts ($c_{i,i,l,k}$) for any individual in that photo as one less than the maximum number of individuals of a single species in the same photo ($c_{i,i,l,k}=\max\left(n_{i,l,k}\right)-1$). This is under the assumption that only one infected individual would be present at a single point in time. Third, we averaged intra‐species contact values across all trapping nights at each site $\left(c_{i,i,l}=\frac{c_{i,i,l,1}+c_{i,i,l,2}+\cdots +c_{i,i,l,K}}{K}\mathrm{for}k=\left\{1,2,\text{…},K\right\}\right)$ to estimate average contacts/trapping night for a single individual of each species at each site. Thus, if a rabid individual entered the population at site *l*, we would assume it would contact $c_{i,i,l}$ individuals of the same species per trapping night.

**TABLE 1:** Parameter values for *R*_0_ calculations

Parameter	Definition	Value	Reference
γ	Per capita rate at which infectious individuals die from rabies	3 days (per 1‐day time step = 1/3)	Hampson et al. (2009)
*P*_rabies|bite_	Probability of rabies given bite in domestic dogs in Tanzania	0.49	Hampson et al. (2009)
*v*	Combined probability of bite with the probability of transmission over that bite when there is contact within and/or between species	0.063, 0.126, 0.25	

We aimed to define inter‐species contacts as the number of individuals of another species that any single individual encountered during each trapping night and, again, used maximum numbers of individuals within a single photo per trapping night counts as proxy. We calculated directional inter‐species contact rates for each species pair, meaning that the number of contacts from species *i* to species *j* at site *l* on night *k* ($c_{i,j,l,k}$) does not necessarily equal the number of contacts from species *j* to species *i* at site *l* on night *k* ($c_{j,i,l,k}$) (Figure 2b). We quantified inter‐species contacts by, first, counting all individuals of each single species ($n_{i}$) and ($n_{j}$) captured together within each single photo and grouping by site (*l*) and night (*k*) into the vectors $n_{i,l,k}$ and $n_{j,l,k}$, respectively. Second, we calculated inter‐species contacts as $c_{i,j,l,k}=\max\left(n_{i,l,k}\right)$and $c_{j,i,l,k}=\max\left(n_{j,l,k}\right)$. Third, we averaged inter‐species contact values across all trapping nights at each site $\left(c_{i,j,l}=\frac{c_{i,j,l,1}+c_{i,j,l,2}+\cdots +c_{i,j,l,K}}{K}\mathrm{and}c_{j,i,l}=\frac{c_{j,i,l,1}+c_{j,i,l,2}+\cdots +c_{j,i,l,K}}{K}\mathrm{for}k=\left\{1,2,\text{…},K\right\}\right)$ to estimate average contacts/trapping night for an individual of species *i* with all individuals of species *j* and vice versa. Thus, if a rabid individual of species *i* entered the population at site *l*, we would assume it would contact $c_{j,i,l}$ individuals of species *j* per recording night *k* and if a rabid individual of species *j* entered the population, it would contact $c_{i,j,l}$ individuals of species *i* per recording night *k*.

### 2.4 *R*_0_ for intra‐species transmission

To determine if species could maintain endemic rabies entirely through intra‐species contacts at these sites, we calculated *R*_0_ for each species and determined if the value was greater than or equal to 1. *R*_0_ is the number of secondary cases produced by each infectious individual in a totally susceptible population. We represented rabies dynamics as a susceptible, exposed, infectious, removed model (Anderson & May, 1992; Keeling & Rohani, 2011) with transmission parameterized as the rate at which individuals in the single‐species population contact each other ($c_{i,i}$), the proportion of the population that is infectious ($p_{i}$), and the combined probability of a bite and transmission of rabies virus via the bite (ν) (Begon et al., 2002) (Table 1). We derived a mathematical expression for *R*_0_ at each site *l* ($R_{0,l}$) by applying the next‐generation method (Diekmann & Heesterbeek, 1990; Heffernan et al., 2005; van den Driessche & Watmough, 2002), such that

(1) $$R_{0,l}=\frac{c_{i,i,l}\nu }{\gamma }.$$

For the step‐by‐step derivation, please see the Supporting Information Appendix 1.

We derived parameter values for $c_{i,i,l}$ from camera trap data as described before and set $\gamma =\frac{1}{3}d^{-1}$ based on the 3‐day infectious period typical for rabies in wildlife (Hampson et al., 2009). To quantify ν, we considered that rabies is maintained by domestic dog populations in Addis Ababa, Ethiopia (Deressa et al., 2010), indicating that $R_{0}\geq 1$ in this population. Setting *R*_0_ = 1 and $c_{i,i,l}$ to the calculated intra‐species contact rate for domestic dogs in Addis Ababa, we calculated the within‐species probability of bite and probability of transmission over that bite (*v*) value for maintenance to be 0.126. We considered this value a mid‐range value and most probable estimate. We calculated an upper bound by multiplying what we felt to be a conservative estimate of the probability of biting given proximity in the camera trap photos (50%) and the probability of rabies virus infection given a bite from an infected dog (49%) (Hampson et al., 2009), which produced an estimated of 0.25. We calculated a lower bound estimate for (*v*) by dividing the (*v*) threshold value for maintenance in domestic dog populations of Addis Ababa by 2, to get a value of 0.063. We then investigated point values (ν={0.063,0.126,0.25}; Table 1) within the range of plausible values [0.063,0.25] to calculate an intra‐species *R*_0_ for each species.

### 2.5 *R*_0_ for inter‐species transmission

If a species cannot maintain rabies transmission through intra‐species contacts, it is possible that a species pair may act as a reservoir and maintain transmission via both intra‐ and inter‐species contacts. To determine if species could maintain endemic rabies entirely through intra‐species and pairwise inter‐species contacts at these sites, we calculated *R*_0_ for each species pair and determined if the value was greater than or equal to 1. Consider species *i* and species *j* at each site *l* – each with a proportion of infected animals, $p_{i,l}$ and $p_{j,l}$ – and an inter‐species contact rate of $c_{i,j,l}$. Let $E_{i,l,t}$ represent the number of new rabies infections that occur in species *i* at each site *l* and timestep *t*, such that

(2) $$E_{i,l,t}=\left(c_{i,i,l}p_{i,l}\nu +c_{i,j,l}p_{j,l}\nu \right)S_{i,l,t-1}.$$

We derived a mathematical *R*_0_ expression for a given species pair at each site *l* (*R*_0, l_) by applying the next‐generation method (Diekmann & Heesterbeek, 1990; Heffernan et al., 2005; van den Driessche & Watmough, 2002) and found

(3) $$R_{0,l}=\frac{1}{2}\frac{\left(c_{1,1,l}\gamma +c_{2,2,l}\gamma \sqrt{c_{1,1,l}^{2}\gamma^{2}-2c_{1,1,l}c_{2,2,l}\gamma^{2}+4c_{1,2,l}c_{2,1,l}\gamma^{2}+c_{2,2,l}^{2}\gamma^{2}}\right)\nu }{\gamma^{2}}.$$

For the full set of equations and the step‐by‐step derivation, please see the Supporting Information Appendix 2.

It was assumed that the probability of bite and the probability of transmission over that bite (*v*) for inter‐species transmission was comparable to intra‐species transmission; thus, in order to determine if each pair of species could maintain rabies, the same range of (*v*) values were used as those used for the intra‐species transmission estimates. Equation (3) will show that maintenance can still be achieved between species if the intra‐species contact rate is high within a species ($c_{i,i,l})$, regardless of contact among species. Because we wanted to capture maintenance when there is both intra‐ and inter‐species transmission, we assumed the *R*_0_ value to be 0 if there was no between‐species contact.

### 2.6 Computing

All analyses were conducted using R, version 4.1.2 (R Core Team, 2021) in RStudio (RStudio Team, 2022). Networks were constructed using the *igraph* package (Csardi & Nepusz, 2006).

## 3 RESULTS

### 3.1 Study sites and wildlife species captured

Slaughter plant facilities had an average area of 4058.7 m^2^ (Table S2). We recorded at each site for an average of five (range of 3–13) trapping nights for a total of 24,362 min (406 h) (Table S2) and 24,512 photographs. Out of the total photographs, 3384 (13.8%) contained images of terrestrial carnivores. We observed seven different species, including domestic dog (*Canis lupus familiaris*), domestic cat (*Felis catus*), spotted hyena (*C. Crocuta*), mongoose (Herpestidae, genus and species undefinable), black‐backed jackal (*Canis mesomelas*), referred to as jackal in this paper, African golden wolf (*C. anthus*) and honey badger (*Mellivora capensis*). Greatest overall abundance was observed for dogs, cats and hyenas (Figure 2). Other animals observed, but not included in the analysis, included cattle, goats, humans and various bird species.

### 3.2 Species distribution and abundance across landscape types

**FIGURE 3:**
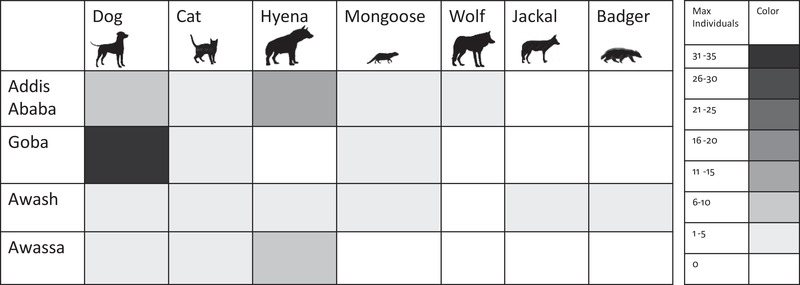
**Terrestrial carnivore species distribution and abundance by site**. The maximum number of individuals observed within a single photo for each species at each site is represented by the greyscale, as indicated in the legend. Greatest species diversity was observed at the Awash and Addis Ababa sites and greatest abundance was observed in dogs and hyenas.

We observed domestic dogs and cats at all sites, hyenas at all but the Goba site, mongoose at all except the Hawassa site, jackals and honey badgers only at the Awash site, and wolves only at the Addis Ababa site (Figure 3). The Goba and Addis Ababa sites had the highest species abundance, measured as having the greatest number of individuals within a single photo, largely due to high numbers of dogs and hyenas, respectively. Observed species diversity was highest at the Awash and Addis Ababa sites and lowest at the rural Goba site. The Addis Ababa site had both high species richness and abundance (Figure 3).

### 3.3 Species abundance throughout the day

**FIGURE 4:**
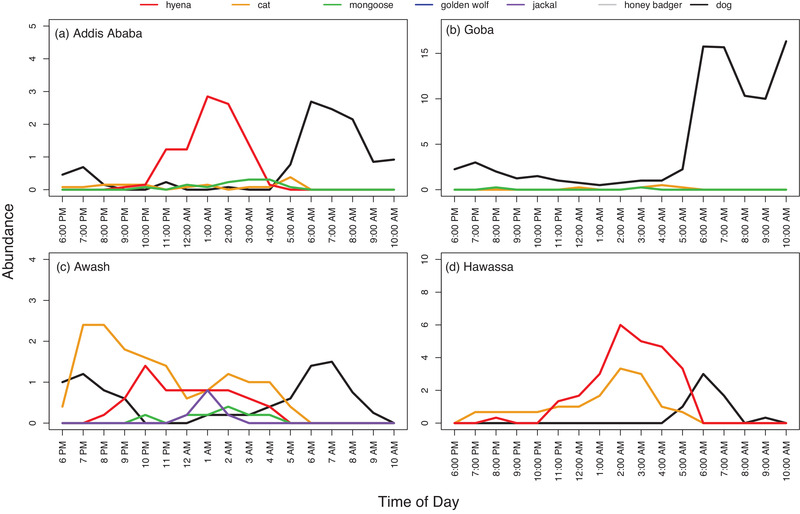
**Temporal trends in species composition and abundance at slaughter plants in four municipalities in Ethiopia**. Averages of maximum individuals per recording period (6:00 PM and 10:00 AM) for each species at each site plotted against time to allow comparison of activity patterns at slaughter plant sites in (a) Addis Ababa, (b) Goba, (c) Awash and (d) Hawassa. Periods where multiple species are active at the same time indicate increased probability of interaction. Each species is represented by a different coloured line as indicated at the top of the figure. Overlapping lines represent species that are active within the same hour at each site. Figures consistently show similar temporal activity trends across sites, with cats and most wildlife species being active at night and dogs active during the day.

Domestic dogs were the only species active during the daytime, with dogs showing peak activity between 5:00 and 10:00 AM. Cats showed peak activity between 2:00 and 5:00 AM and again at 6:00 PM. We observed all wildlife species, including hyenas, mongoose, black‐backed jackals, wolves and honey badgers, between the hours of 7:00 PM and 5:00 AM, indicating that the wildlife species observed displayed largely nocturnal behaviour. Cats were also regularly observed throughout the night (Figure 4).

Multiple species were active between the hours of 10:00 PM and 5:00 AM. At the Addis Ababa site, five species were active between 2:00 and 5:00 AM, including dogs, cats, hyenas, mongoose and wolves. At the Goba site, dogs and cats were active between 12:00 and 5:00 AM, with the occasional appearance of a single mongoose at 3:00 AM and again at 8:00 PM. In Awash, six species were active between 12:00 and 3:00 AM, including dogs, cats, hyenas, mongoose, jackals and honey badgers. At Hawassa, cats and hyenas were consistently active between 7:00 PM and 6:00 AM (Figure 4). Overall, activity patterns among wildlife species remained consistent across sites (Tables S3–S6; Figures S1–S4). Though there was some brief overlap, dogs and hyenas revealed nearly opposite activity patterns which could suggest avoidance behaviour (Figure 4, Table S7).

### 3.4 Intra‐species contacts and *R*_0_

We predicted the number of secondary cases produced by each primary case in a totally susceptible population (*R*_0_) if rabies were to circulate among these wildlife communities to determine which species could maintain rabies ($R_{0}\geq 1$) solely from intra‐species contacts at a range of plausible ν values. This parameter (ν) represented the probability of biting and subsequent transmission among individuals sighted in the same camera trap photo. At our most likely and mid‐range transmission estimates (ν=0.126), dogs showed potential for maintenance at the Addis Ababa and Goba sites, whereas hyenas showed potential for maintenance at the Addis Ababa and Hawassa sites (Table 2). At the lowest transmission value (ν=0.063), dogs only showed potential for maintenance at the Goba site and hyenas only showed potential for maintenance at the Hawassa site (Table S8). At the highest transmission value (ν=0.25), dogs showed potential for maintenance at an additional site (Hawassa) and cats showed potential at the Awash and Hawassa sites. Overall, the highest predicted *R*_0_ values were calculated for domestic dog populations at the Goba slaughter plant site and rabies maintenance could potentially be achieved by the most species at the Hawassa slaughter plant site. Intra‐species interactions were too low for rabies maintenance in mongoose and golden wolves at the Addis Ababa site and honey badgers at the Awash site (Table S8).

**TABLE 2:** Intra‐species *R*_0_ calculations at the midrange transmission probability (ν= 0.126)

	Species					
Site	Dog	Cat	Hyena	Mongoose	Wolf	Badger
Addis Ababa	1^a^	0.06	1.32^a^	0.03	0.06	0
Goba	6.78^a^	0	0	0	0	0
Awash	0.38	0.69	0.15	0	0	0.08
Hawassa	0.76	0.89	2.04^a^	0	0	0

^a^ Potential for rabies maintenance (predicted $R_{0}\geq 1$).

### 3.5 Inter‐species contacts and *R*_0_

**FIGURE 5:**
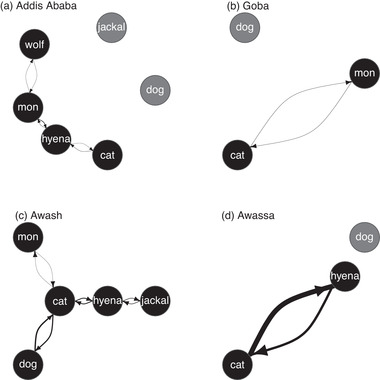
**Inter‐species contact networks**. Averages of maximum inter‐species interactions observed between each species pair per recording period (6:00 PM and 11:00 AM) at each site were placed into a matrix and used to create contact networks for the (a) Addis Ababa, (b) Goba, (c) Awash and (d) Hawassa sites. Each node represents the species labelled, and each weighted directional edge represents the number of inter‐species contacts. The most inter‐species interactions were observed for hyenas and cats (a, c, d). Other notable species pairs that were observed in the same camera trap photo included mongoose and hyenas (a), dogs and cats (c), mongoose and cats (c), and jackals and hyenas (c).

The inter‐species contact networks for each site (Figure 5, Tables S9–S12) show that the most inter‐species contacts occur between hyenas and cats at the Addis Ababa, Awash and Hawassa sites, with cats having more opportunity to bite hyenas than hyenas to bite cats in Addis Ababa and Hawassa. Other notable interspecies interactions included contacts between mongoose and hyenas at the Addis Ababa site, with mongoose having more opportunity to bite hyenas because hyenas are more abundant, contacts between dogs and cats at the Awash site, with dogs having more opportunity to bite cats than cats to bite dogs because cats are more abundant, and equal contacts between mongoose and cats in both Goba and Awash, mongoose and wolves in Addis Ababa, and jackals and hyenas in Awash. The Awash site had the greatest number of inter‐species interactions, whereas the Hawassa site had the strongest interactions (cat–hyena).

Using the contact data, we predicted the number of secondary cases produced by each primary case in a totally susceptible population (*R*_0_) if rabies were to circulate among these wildlife communities to determine which species could maintain rabies ($R_{0}\geq 1$) with intra‐ and inter‐species contacts at a range of plausible ν values. This parameter (ν) represented the probability of biting and subsequent transmission among individuals sighted in the same camera trap photo. Consistent with contact network observations, at the midrange transmission value (*v* = 0.126), potential for rabies maintenance could occur between hyena and cat populations at the Addis Ababa and Hawassa sites and almost at the Awash site (*R*_0_ = 0.99), between dogs and cats at the Awash site, and between hyenas and mongoose in Addis Ababa (Table 3). At the highest transmission value (*v* = 0.25), hyenas and cats showed potential for maintenance at an additional site (Awash), and mongoose and cats showed potential for maintenance in Awash (Table S13). At the low transmission value (*v* = 0.063), hyenas and cats still showed potential for maintenance at the Hawassa site (Table S13). Though notable interaction occurred between hyenas and jackals in Awash and wolves and mongoose in Addis Ababa, *R*_0_ maintenance values were not predicted. The highest overall inter‐species *R*_0_ values were predicted between hyenas and cats at the Hawassa site, while the greatest number of different species pairs was observed at the Awash site (Table 3, Table S13).

**TABLE 3:** Combined inter‐ and intra‐species *R*_0_ calculations at the midrange transmission probability (ν= 0.126)

	Species					
Site	Dog–cat	Cat–spotted hyena	Black‐backed jackal–spotted hyena	Hyena–mongoose	Wolf–mongoose	Mongoose–cat
Addis Ababa	0	1.38^a^	0	1.37^a^	0.1	0
Goba	0	0	0	0	0	0.1
Awash	1.32^a^	0.99	0.32	0	0	0.7
Hawassa	0	3.68^a^	0	0	0	0

^a^ Potential for rabies maintenance (predicted $R_{0}\geq 1$).

## 4 DISCUSSION

In this study, we used contact structure as a proxy for rabies transmission potential to identify wildlife species in Ethiopia with the highest likelihood of maintaining the virus, either as a single species or a species pair, given introduction into closed populations located at sites with high contact opportunity. Knowledge of all potential reservoir species is essential for the control of any viral zoonotic disease, considering the potential for the amplification of transmission in populations where the pathogen already exists as well as the potential for establishing new reservoir populations. Our study suggests that multi‐species host communities for rabies transmission could exist in Ethiopia. Within these communities, wild urban carnivores, primarily hyenas, may pose a risk for independent maintenance. Though highest rates of inter‐species contact were observed between hyenas and cats, cats might serve as dead‐end‐hosts for rabies transmission (Lembo et al., 2008). If this holds true in Ethiopia, between‐species maintenance would only be able to be achieved between hyena and mongoose at a single site. However, cats have demonstrated effective transmission of mongoose rabies in South Africa (Grobbelaar et al., 2020), indicating the need for further investigation at the molecular level to clarify the role that cats may play in Ethiopia. The probability of maintenance may be further reduced by prey species withdrawing from predator species as opposed to showing aggression, despite behavioural changes that may occur due to rabies infection. However, cats have demonstrated effective transmission of mongoose rabies in South Africa (Grobbelaar et al., 2020), indicating the need for further investigation at the molecular level to clarify the role that cats may play in Ethiopia.

Specific sites – namely, Addis Ababa and Hawassa – were identified as high risk for both intra‐ and inter‐species transmission. Addis Ababa is one of the largest cities in Africa, with a population density of more than 3.7 million people (Ethiopian Central Statistics Agency, 2021) and producing roughly 1 billion kg of waste/year, much of which remains uncontained and available for scavengers (Desta et al., 2014). This environment is optimal for urban carnivores, especially hyenas, a species known to survive almost entirely off of anthropogenic food sources (Yirga et al., 2013). Hawassa hosted larger populations of the key species identified as the most likely to be able to maintain transmission either independently (hyenas) or as a pair (hyena–cat), making it a critical area for further investigation. Our results indicate that urban and peri‐urban areas with an abundance of waste may serve as areas for greater rabies transmission risk compared to more rural areas.

Our study had several limitations. We relied on a model where the probability of transmission given contact is constant and outcomes co‐vary in proportion to contacts (Begon et al., 2002; Manlove et al., 2017). However, it is still important to note that true transmission potential is dependent on more than contact structure alone. There are many other critical variables that affect probability of contact and probability of transmission over that contact such as seasonal, environmental, genetic, viral, immunological and behavioural factors (Klepac et al., 2009; Pomeroy et al., 2019; VanderWaal & Ezenwa, 2016; White et al., 2018) that are beyond the scope of our investigation and not incorporated in our calculations. Moreover, our contact rate estimates might be inaccurate. We quantified the model with contacts from supplemental foraging sites, but in reality, within‐species contacts when animals are not at these sites might differ. Contact behaviour of a rabid animal would be heavily influenced by whether an individual is affected by the ‘furious’ or the ‘dumb’ form of rabies. We were also unable to distinguish individuals in camera trap photos which introduced the risk of pseudo‐replication; thus, consecutive repeat contacts could inflate contact estimates. To adjust for this, we used maximum numbers of individuals of each species within a photo per trapping night and averaged those values across all trapping nights for each site to ensure that interactions were independent events throughout each recording period (Richomme et al., 2006). However, we acknowledge that there were likely more contacts between new individuals throughout the recording period than could be captured within a single photo. We were also limited in the amount of within‐ and between‐site variation that we could capture due to sampling being limited to four sites over 3–13 trapping nights during the rainy season. We selected sites that were comparable both in size and slaughter practices while also trying to capture as much natural variability as possible and sampled intensively (1‐min intervals) over each available trapping night, and selected sites to be as representative of multiple landscape types across different regions as possible through discussions with local municipalities. Nevertheless, this introduces uncertainty in the consistency of our contact rate estimates.

Our species contact estimates were specific to supplemental foraging sites, similar to other studies of rabies transmission among wildlife (Hirsch et al., 2013; Macdonald et al., 2004; Totton et al., 2002). Because supplemental foraging sites provide increased opportunity for both intra‐ and inter‐specific contacts (Totton et al., 2002), our estimates provide an upper bound for contact rates throughout Ethiopia. We assume that if a species or species pair cannot achieve maintenance under these highest contact conditions, it is unlikely they would be able to achieve maintenance with a more homogeneous distribution of lower contact frequency.

Our findings illustrate the complex relationship between humans and hyenas. In natural environments, spotted hyenas exist in fission–fusion societies in which they spend most of their time in subgroups and only come together as a larger group, or clan, for important events such as territorial defence (Holekamp et al., 1997). Urbanization in Ethiopia has resulted in reduced hyena social structure, allowing for the existence of large groups of individuals with more frequent interaction and thus, more potential for rabies transmission (Schramme, 2015). Other large carnivores show similar responses in densely populated urban settings, including coyotes (*Canis latrans*) in North America, leopards (*Panthera pardus*) in India, brown bears (*Ursus arctos*) in Eastern Europe and jaguarondi (*Puma yagouaroundi*) in South America (Young et al., 2020). Yet, these urban carnivores provide critical waste clearing services (Braczkowski et al., 2018; Yirga et al., 2013), digesting 18 kg food/h (Smith & Holekamp, 2010), including every part of an animal except hair and hooves (Yirga et al., 2015), which can reduce sources of pathogen contamination and spread in the environment. Leopards living in Mumbai could prevent up to 90 human rabies deaths a year due to predation on free roaming dogs (Braczkowski et al., 2018), a role that hyenas may play in Ethiopia as well. These findings suggest that hyenas have the potential to both increase and decrease rabies transmission and the actual effect depends on ecological and anthropogenic contexts.

Our findings have implications for waste management. Any uncontained waste disposal practices have the potential to promote supplemental scavenging by large groups of urban carnivores. At the household level, community education about appropriate backyard waste disposal may help limit species interactions. It has been suggested that feeding scraps to dogs during the day rather than throwing them out at night may limit interactions with hyenas in the Serengeti (Craft et al., 2017). Some municipalities in Ethiopia have already started to build barriers around their waste disposal facilities to prevent such activity. However, if no waste resources are available to urban carnivores, there is potential for increased rates of livestock raiding and more rapid accumulation of waste due to reduced disposal capacity that is generally provided by the urban carnivores (Newsome & Van Eeden, 2017). Communication across all government sectors will be essential to establish a balance that can reduce the potential for rabies transmission while also managing city waste and maintaining healthy wildlife populations. Management strategies and control measures must be tailored to specific localities and times of day based on our findings regarding differences in reservoir species, temporal habits and rabies maintenance capacity across multiple sites.

Our findings also have implications for the understanding of multi‐species disease reservoirs. We found that rabies maintenance potential was underestimated when only within‐species contacts were considered. When both within‐ and between‐contacts were considered, we predict maintenance at a site that was not considered at risk for rabies maintenance when only within‐species contacts were observed. Results from this study cam also inform transmission of other priority pathogens such as distemper and parvovirus in wild carnivore populations, especially where there is spillover from domestic dogs and cats to endangered populations such as the Ethiopian wolf (Haydon et al., 2002). Our spatial and temporal data show that the potential for indirect pathogen transmission could also be very high, depending on pathogen viability in the environment. For example, the presence of *Salmonella*, some of which may carry anti‐microbial resistance genes, can persist in the soil for at least 21 days (Jechalke et al., 2019). Though there were very few direct contacts between hyenas and dogs, if a dog were to leave behind *Salmonella*‐infected faecal waste, the hyenas that arrive shortly after and scavenge around the same areas would have a high risk of exposure. If exposure results in infection, the hyenas could then carry those *Salmonella* strains and/or anti‐microbial resistance genes and spread them throughout the environment, especially to other wildlife populations.

This study is a first step towards understanding the role that wild carnivore populations play as potential reservoirs for rabies maintenance in Ethiopia, highlighting the role of intra‐ and inter‐species contacts and identifying priority species and locations for future study. However, we must emphasize the role that dogs play as primary reservoirs for rabies transmission in Ethiopia. As control continues to progress in the dog population, a further study of the role that hyenas in urban and peri‐urban areas of Ethiopia play as reservoirs for rabies transmission should become increasingly important. Though hyenas were identified as a priority species for further investigation, the role of other wildlife species should not be overlooked. Evidence from molecular epidemiological data from rabid animals throughout Ethiopia suggests the potential for the existence of a divergent RABV lineage in side‐striped jackals in the southern region of the country (Binkley et al., 2021). In South Africa, it is known that mongoose harbour an independent lineage of the virus (Nel et al., 2005), whereas black‐backed jackals (Zulu et al., 2009) and bat‐eared foxes (Sabeta et al., 2007) may be showing the beginnings of divergent lineages in certain parts of the country. Detailed epidemiological data on the prevalence of rabies in these wild carnivore populations in urban and peri‐urban areas in Ethiopia, along with many other pathogens, remain largely unknown due to a lack of ongoing wildlife disease surveillance. This information will be critical to further clarify the role that these species play as reservoirs for rabies transmission. Once more data become available, it will be important to generate rabies maintenance models that can look at the potential for more than two species to maintain rabies independent of domestic dog populations within a maintenance community. This will be critical for understanding the full scope of rabies transmission in Ethiopia, especially during later phases of elimination when reductions in dog rabies reach significant levels. Such models will allow us to predict which species to target and what methods may be most effective to prevent establishment and/or propagation of such maintenance communities. Our methods can also be applied to the investigation of other pathogens to improve understanding of transmission ecology.

## AUTHOR CONTRIBUTIONS

Laura Binkley: conceptualization, methodology, validation, formal analysis, investigation, resources, data curation, writing‐original draft, writing‐review and editing, visualization, project administration, funding acquisition; Jeanette O'Quin: conceptualization, methodology, resources, writing‐review and editing, supervision; Balbine Jourdan: methodology, investigation, writing‐review and editing; Getnet Yimer: resources, writing‐review and editing; Asefa Deressa: resources, writing‐review and editing; Laura W. Pomeroy: conceptualization, methodology, formal analysis, writing‐review and editing, visualization, project administration, funding acquisition, supervision, software.

## CONFLICTS OF INTEREST

All authors declare that they have no conflicts of interest.

## ETHICS STATEMENT

The authors confirm that the ethical policies detailed on the journal's author guidelines page have been followed and the appropriate ethical review approval was obtained. We obtained permitting and permission from all necessary administrative bodies, including the Ethiopian Wildlife Conservation Authority, the Ethiopian Public Health Institute and the Ethiopian Broadcasting Authority. We met with the local municipality, local authorities and slaughter plant owners in each of study sites and surrounding areas to explain the study and gain approval to conduct the study.

## Supporting information



Supporting Information

## Data Availability

The authors confirm that the data supporting the findings of this study are available within the article [and/or] its supplementary materials. Raw data are available on request from the corresponding author.
