# Supplementary material for: Quantifying intra‐ and inter‐species contact rates at supplemental feeding sites in Ethiopia to inform rabies maintenance potential of multiple host species
Source: Transbound Emerg Dis. 2022 Nov 15;69(6):3837–49. doi: 10.1111/tbed.14755 (PMC10099229; doi:10.1111/tbed.14755)
Supplement: Supplementary file 1 — Supporting Information [file TBED-69-3837-s001.pdf]

Supplementary information for

Quantifying intra- and inter-species contact rates at supplemental feeding sites in Ethiopia to inform rabies maintenance potential of multiple host species

Laura Binkley[1,2]*, Jeanette O'Quin[1], Balbine Jourdan[3], Getnet Yimer[2], Laura W. Pomeroy[2,3]

1 Department of Veterinary Preventive Medicine, College of Veterinary Medicine, The Ohio State University, Columbus, Ohio, USA

2 Global One Health initiative, Office of International Affairs, The Ohio State University, Columbus, Ohio, USA

3 College of Veterinary Medicine, The Ohio State University, Columbus, Ohio, USA

4 Rabies and Other Zoonotic Diseases Research Division, Ethiopian Public Health Institute, Addis Ababa, Ethiopia

5 Environmental Health Sciences, College of Public Health, The Ohio State University, Columbus, Ohio, USA

6 Translational Data Analytics Institute, The Ohio State University, Columbus, Ohio, USA

* corresponding author (binkley.69@osu.edu)

This pdf includes:

Appendix 1	Modeling rabies transmission within species
Appendix 2	Finding R_0 for the within species transmission model
Table S1	Study sites by urban-rural population and highland/lowland
Table S2	Area (in m^2) and total recording time (in minutes) for all study sites
Table S3	Temporal trends in species composition and abundance at slaughter plants in Addis Ababa
Table S4	Temporal trends in species composition and abundance at slaughter plants in Goba
Table S5	Temporal trends in species composition and abundance at slaughter plants in Awash
Table S6	Temporal trends in species composition and abundance at slaughter plants in Hawassa

Table S7 Species observed during same hour but never observed together

Table S8 Intra-species contact rates at all *v* threshold values.

Table S9 Contacts at Addis Ababa with potential transmission from the species listed horizontally to those listed vertically

Table S10 Contacts at Goba with potential transmission from the species listed horizontally to those listed vertically.

Table S11 Contacts at Awash with potential transmission from the species listed horizontally to those listed vertically.

Table S12 Contacts at Hawassa with potential transmission from the species listed horizontally to those listed vertically.

Table S13 Inter-species contact rates at all *v* threshold values.

Figure S1 Temporal trends in species composition and abundance at slaughter plants in Addis Ababa with standard error bars

Figure S2 Temporal trends in species composition and abundance at slaughter plants in Goba with standard error bars

Figure S3 Temporal trends in species composition and abundance at slaughter plants in Awash with standard error bars

Figure S4 Temporal trends in species composition and abundance at slaughter plants in Hawassa with standard error bars

Appendix 1: finding R_0 for the within species transmission model

We consider the SEIR model to represent rabies transmission within a single species. This model divides the population into four disease states: susceptible (S) individuals, exposed (E) individuals who are infected but not yet infectious, infectious (I) individuals, and individuals that are removed (R) from population and transmission dynamics because they have died due to rabies infection. In order to parameterize the model following *Begon et al. 2002*, let c represent the rate at which any individuals in the population contact each other, let p represent the proportion of the population that is infectious, and let ν represent the probability that contact with an infectious individual will result in a new infection in the susceptible individual participating in that contact. Then, the susceptible category changes according to

$$\frac{dS}{dt} = -cp\nu S = \text{-}c\nu \frac{I}{N} S \tag{1}$$

when p is assumed to be $\frac{I}{N}$. Let σ represent the rate at which exposed individuals become infectious and let γ represent the rate at which infectious individuals die due to rabies. Then,

$$\frac{dE}{dt} = c\nu \frac{I}{N} S - \sigma E \tag{2}$$

$$\frac{dI}{dt} = \sigma E - \gamma I \tag{3}$$

$$\frac{dR}{dt} = \gamma I \tag{4}$$

Following *van den Dressche and Watmough 2002* and *Heffernan et al. 2002*, we can find an expression for R_0 using the next generation method. Let $\mathbf{X} = (x_1 + ... + x_m + ...x_n)$ represent n disease states where the first m compartments represent the infected and infectious states so that, for our SEIR model with four disease states and two infected and infectious states,

$$\mathbf{X} = \begin{bmatrix} E \\ I \\ S \\ R \end{bmatrix}. \tag{5}$$

Let $\mathbf{F_i}(\mathbf{X})$ represent the"rate of appearance of new infections into compartment i", $\mathbf{V_i^+}(\mathbf{X})$ represent the "rate of transfer of individuals into compartment i by all other means," and $\mathbf{V_i^-}(\mathbf{X})$ represent the "rate of transfer of individuals out of compartment i", so that $\mathbf{V_i}(\mathbf{X}) = \mathbf{V_i^-}(\mathbf{X}) - \mathbf{V_i^+}(\mathbf{X})$. Since new infections are only apparent in E,

$$\mathbf{F_i}(\mathbf{X}) = \begin{bmatrix} c\nu \frac{I}{N} S \\ 0 \end{bmatrix} \tag{6}$$

and

$$\mathbf{V_i}(\mathbf{X}) = \begin{bmatrix} \sigma E \\ \gamma I - \sigma E \end{bmatrix}. \tag{7}$$

For $i, j = 1, ..., m$, let

$$\mathbf{F}(\mathbf{X}) = \left[\frac{\partial \mathbf{F_i}(\mathbf{X})}{\partial \mathbf{x_j}} x_0 \right] \tag{8}$$

and

$$\mathbf{V} = \left[\frac{\partial \mathbf{V_i}(\mathbf{X})}{\partial \mathbf{x_j}} x_0 \right] \tag{9}$$

such that

$$\mathbf{F}(\mathbf{X}) = \begin{bmatrix} 0 & c\nu S^*/N \\ 0 & 0 \end{bmatrix} \tag{10}$$

and

$$\mathbf{V}(\mathbf{X}) = \begin{bmatrix} \sigma & 0 \\ -\sigma & \gamma \end{bmatrix}. \tag{11}$$

At the disease-free equilibrium, x_0, $E = I = 0$, so that

$$\mathbf{X} = \begin{bmatrix} 0 & 0 & S^* & 0 \end{bmatrix}' = \begin{bmatrix} 0 & 0 & N & 0 \end{bmatrix}' \tag{12}$$

and $\mathbf{F}(\mathbf{X})$ and $\mathbf{V}(\mathbf{X})$ evaluated at the disease-free equilibrium can be written as

$$\mathbf{F}(\mathbf{X}) = \begin{bmatrix} 0 & c\nu \\ 0 & 0 \end{bmatrix} \tag{13}$$

and

$$\mathbf{V}(\mathbf{X}) = \begin{bmatrix} \sigma & 0 \\ -\sigma & \gamma \end{bmatrix}. \tag{14}$$

The inverse matrix $\mathbf{V^{-1}}$ is

$$\mathbf{V^{-1}} = \begin{bmatrix} \frac{\gamma}{\sigma\gamma} & 0 \\ \frac{\sigma}{\sigma\gamma} & \frac{\sigma}{\sigma\gamma} \end{bmatrix} = \begin{bmatrix} \frac{1}{\sigma} & 0 \\ \frac{1}{\gamma} & \frac{1}{\gamma} \end{bmatrix} \tag{15}$$

Let $\mathbf{FV^{-1}}$ denote the next generation matrix, so that

$$\mathbf{FV^{-1}} = \begin{bmatrix} 0 & c\nu \\ 0 & 0 \end{bmatrix} \begin{bmatrix} \frac{1}{\sigma} & 0 \\ \frac{1}{\gamma} & \frac{1}{\gamma} \end{bmatrix} = \begin{bmatrix} \frac{c\nu}{\gamma} & \frac{c\nu}{\gamma} \\ 0 & 0 \end{bmatrix}. \tag{16}$$

To find the dominant eigenvalue, which denotes R_0, we first write the characteristic equation

$$|\mathbf{FV^{-1}} - \lambda\mathbf{I}| = \begin{bmatrix} \frac{c\nu}{\gamma} & \frac{c\nu}{\gamma} \\ 0 & 0 \end{bmatrix} - \begin{bmatrix} \lambda & 0 \\ 0 & \lambda \end{bmatrix} = 0 \tag{17}$$

$$\det \begin{bmatrix} \frac{c\nu}{\gamma} - \lambda & \frac{c\nu}{\gamma} \\ 0 & -\lambda \end{bmatrix} = \lambda^2 - \frac{\lambda c\nu}{\gamma} \tag{18}$$

There are two eigenvalues: $\lambda_1 = 0$ and $\lambda_2 = \frac{c\nu}{\gamma}$. R_0 is represented by the dominant eigenvalue, so

$$R_0 = \frac{c\nu}{\gamma} \tag{19}$$

Appendix 2: finding R_0 for the within and between species transmission model

We also consider the SEIR model to represent rabies transmission within **and between** pairs of species. This model divides the population into four disease states: susceptible (S) individuals, exposed (E) individuals who are infected but not yet infectious, infectious (I) individuals, and individuals that are removed (R) from population and transmission dynamics because they have died due to rabies infection. In order to parameterize the model following *Begon et al. 2002*, let $c_{i,i}$ represent the within species contacts for species i, let $c_{i,j}$ represent the contact rate between species i and species j, and let p_j represent the proportion of the population j that is infectious. We also designate the parameter ν, which represents the probability that contact with an infectious individual will result in a new infection in the susceptible individual participating in that contact, which we assume is the same across all species. Then, the susceptible category changes according to

$$\frac{dS_i}{dt} = -(c_{i,i}p_i + c_{i,j}p_j)\nu S_i = -(c_{i,i}\frac{I_i}{N_i} + c_{i,j}\frac{I_j}{N_j})\nu S_i \tag{20}$$

where p_i is assumed to be $\frac{I_i}{N_i}$ and p_j is assumed to be $\frac{I_j}{N_j}$. Let σ represent the rate at which exposed individuals become infectious and let γ represent the rate at which infectious individuals die due to rabies, both of which we assume is the same across all species. Then,

$$\frac{dE_i}{dt} = (c_{i,i}\frac{I_i}{N_i} + c_{i,j}\frac{I_j}{N_j})\nu S_i - \sigma E_i \tag{21}$$

$$\frac{dI_i}{dt} = \sigma E_i - \gamma I_i \tag{22}$$

$$\frac{dR_i}{dt} = \gamma I_i \tag{23}$$

Following *van den Dressche and Watmough 2002* and *Heffernan et al. 2002*, we can find an expression for R_0 using the next generation method. Let $\mathbf{X} = (x_1 + ... + x_m + ...x_n)$ represent n disease states where the first m compartments represent the infected and infectious states so that, for our SEIR model with four disease states and two infected and infectious states,

$$\mathbf{X} = \begin{bmatrix} E_1 \\ E_2 \\ I_1 \\ I_2 \\ S_1 \\ S_2 \\ R_1 \\ R_2 \end{bmatrix}. \tag{24}$$

Let $\mathbf{F_i}(\mathbf{X})$ represent the"rate of appearance of new infections into compartment i", $\mathbf{V_i^+}(\mathbf{X})$ represent the "rate of transfer of individuals into compartment i by all other means," and $\mathbf{V_i^-}(\mathbf{X})$ represent the "rate of transfer of individuals out of compartment i", so that $\mathbf{V_i}(\mathbf{X}) = \mathbf{V_i^-}(\mathbf{X}) - \mathbf{V_i^+}(\mathbf{X})$. Since new infections are only apparent in E_1 and E_2,

$$\mathbf{F_i}(\mathbf{X}) = \begin{bmatrix} c_{1,1}\frac{I_1}{N_1}\nu S_1 + c_{1,2}\frac{I_2}{N_2}\nu S_1 \\ c_{2,1}\frac{I_1}{N_1}\nu S_2 + c_{2,2}\frac{I_2}{N_2}\nu S_2 \\ 0 \\ 0 \end{bmatrix} \tag{25}$$

and

$$\mathbf{V_i}(\mathbf{X}) = \begin{bmatrix} \sigma E_1 \\ \sigma E_2 \\ \gamma I_1 - \sigma E_1 \\ \gamma I_2 - \sigma E_2 \end{bmatrix}. \tag{26}$$

For $i, j = 1, ..., m$, let

$$\mathbf{F}(\mathbf{X}) = \left[\frac{\partial \mathbf{F_i}(\mathbf{X})}{\partial \mathbf{x_j}} x_0\right] \tag{27}$$

and

$$\mathbf{V} = \left[\frac{\partial \mathbf{V_i}(\mathbf{X})}{\partial \mathbf{x_j}} x_0\right] \tag{28}$$

such that

$$\mathbf{F}(\mathbf{X}) = \begin{bmatrix} 0 & 0 & c_{1,1}\nu\frac{S_1^*}{N_1} & c_{1,2}\nu\frac{S_1^*}{N_2} \\ 0 & 0 & c_{2,1}\nu\frac{S_2^*}{N_1} & c_{2,2}\nu\frac{S_2^*}{N_2} \\ 0 & 0 & 0 & 0 \\ 0 & 0 & 0 & 0 \end{bmatrix} \tag{29}$$

and

$$\mathbf{V}(\mathbf{X}) = \begin{bmatrix} \sigma & 0 & 0 & 0 \\ 0 & \sigma & 0 & 0 \\ -\sigma & 0 & \gamma & 0 \\ 0 & -\sigma & 0 & \gamma \end{bmatrix}. \tag{30}$$

At the disease-free equilibrium, x_0, $E = I = 0$, so that

$$\mathbf{X} = \begin{bmatrix} 0 \\ 0 \\ 0 \\ 0 \\ S_1^* \\ S_2^* \\ 0 \\ 0 \end{bmatrix} = \begin{bmatrix} 0 \\ 0 \\ 0 \\ 0 \\ N_1 \\ N_2 \\ 0 \\ 0 \end{bmatrix} \tag{31}$$

and $\mathbf{F}(\mathbf{X})$ and $\mathbf{V}(\mathbf{X})$ evaluated at the disease-free equilibrium can be written as

$$\mathbf{F}(\mathbf{X}) = \begin{bmatrix} 0 & 0 & c_{1,1}\nu & c_{1,2}\nu\frac{N_1}{N_2} \\ 0 & 0 & c_{2,1}\nu\frac{N_2}{N_1} & c_{2,2}\nu \\ 0 & 0 & 0 & 0 \\ 0 & 0 & 0 & 0 \end{bmatrix} \tag{32}$$

and

$$\mathbf{V}(\mathbf{X}) = \begin{bmatrix} \sigma & 0 & 0 & 0 \\ 0 & \sigma & 0 & 0 \\ -\sigma & 0 & \gamma & 0 \\ 0 & -\sigma & 0 & \gamma \end{bmatrix}. \tag{33}$$

We calculate the inverse matrix $\mathbf{V^{-1}}$ by Gauss-Jordan elimination, taking six steps. First, start on row 3 and add row 1. Second, on row 3, multiply by $\frac{1}{\gamma}$. Third, move to row four and add row

2. Fourth, on row four, multiply it by $\frac{1}{\gamma}$. Fifth, move to row 1 and multiply it by $\frac{1}{\sigma}$. Sixth, move to row 2 and multiply it by $\frac{1}{\sigma}$. Then,

$$\mathbf{V^{-1}} = \begin{bmatrix} \frac{1}{\sigma} & 0 & 0 & 0 \\ 0 & \frac{1}{\sigma} & 0 & 0 \\ \frac{1}{\gamma} & 0 & \frac{1}{\gamma} & 0 \\ 0 & \frac{1}{\gamma} & 0 & \frac{1}{\gamma} \end{bmatrix}. \tag{34}$$

Let $\mathbf{FV^{-1}}$ denote the next generation matrix, so that

$$\mathbf{FV^{-1}} = \begin{bmatrix} 0 & 0 & c_{1,1}\nu & c_{1,2}\nu\frac{N_1}{N_2} \\ 0 & 0 & c_{2,1}\nu\frac{N_2}{N_1} & c_{2,2}\nu \\ 0 & 0 & 0 & 0 \\ 0 & 0 & 0 & 0 \end{bmatrix} \begin{bmatrix} \frac{1}{\sigma} & 0 & 0 & 0 \\ 0 & \frac{1}{\sigma} & 0 & 0 \\ \frac{1}{\gamma} & 0 & \frac{1}{\gamma} & 0 \\ 0 & \frac{1}{\gamma} & 0 & \frac{1}{\gamma} \end{bmatrix} = \begin{bmatrix} \frac{c_{1,1}\nu}{\gamma} & \frac{c_{1,2}\nu}{\gamma}\frac{N_1}{N_2} & \frac{c_{1,1,}\nu}{\gamma} & \frac{c_{1,2}\nu}{\gamma}\frac{N_1}{N_2} \\ \frac{c_{2,1}\nu}{\gamma}\frac{N_2}{N_1} & \frac{c_{2,2}\nu}{\gamma} & \frac{c_{2,1}\nu}{\gamma}\frac{N_2}{N_1} & \frac{c_{2,2}\nu}{\gamma} \\ 0 & 0 & 0 & 0 \\ 0 & 0 & 0 & 0 \end{bmatrix}. \tag{35}$$

To find the dominant eigenvalue, which denotes R_0, we first write the characteristic equation

$$|\lambda - \mathbf{FV^{-1}I}| = \begin{bmatrix} \lambda - \frac{c_{1,1}\nu}{\gamma} & \frac{c_{1,2}\nu}{\gamma}\frac{N_1}{N_2} & \frac{c_{1,1,}\nu}{\gamma} & \frac{c_{1,2}\nu}{\gamma}\frac{N_1}{N_2} \\ \frac{c_{2,1}\nu}{\gamma}\frac{N_2}{N_1} & \lambda - \frac{c_{2,2}\nu}{\gamma} & \frac{c_{2,1}\nu}{\gamma}\frac{N_2}{N_1} & \frac{c_{2,2}\nu}{\gamma} \\ 0 & 0 & \lambda - 0 & 0 \\ 0 & 0 & 0 & \lambda - 0 \end{bmatrix} = 0 \tag{36}$$

$$\lambda^4 - \frac{\nu(c_{1,1} + c_{2,2})\lambda^3}{\gamma} + \frac{\nu^2(c_{1,1}c_{2,2} - c_{1,2}c_{2,1})\lambda^2}{\gamma^2} = 0 \tag{37}$$

There are two eigenvalues. R_0 is represented by the dominant eigenvalue, but this will depend on the magnitude of the contact rates within and between species. So,

$$R_0 = \frac{1}{2}\frac{(c_{1,1}\gamma + c_{2,2}\gamma\sqrt{c_{1,1}^2\gamma^2 - 2c_{1,1}c_{2,2}\gamma^2 + 4c_{1,2}c_{2,1}\gamma^2 + c_{2,2}^2\gamma^2})\nu}{\gamma^2} \tag{38}$$

Table S1. Study sites by urban-rural population and highland/lowland

	**Landscape**		
**Site**	**Urban**	**Rural**	**Highland/Lowland**
**Addis Ababa**	3,433, 999	-	Highland
**Goba**	-	51,836	Highland
**Awash**	31,437	15,472	Lowland
**Hawassa**	335,508	120,150	Lowland

Source: https://www.statsethiopia.gov.et/wp-content/uploads/2019/05/Population-Projection-At-Wereda-Level-from-2014-2017.pdf

Table S2. Area (in m^2) and total recording time (in minutes) for all study sites

**Study Site**	**Area m^2**	**Total recording time (min)**
**Addis Ababa**	3154.61	13070
**Goba**	4502.49	3689
**Awash**	4241.6	4633
**Hawassa**	4336.31	2970

Table S3. Temporal trends in species composition and abundance at slaughter plants in Addis Ababa

Dog

	Period															
**Hour**	**1**	**2**	**3**	**4**	**5**	**6**	**7**	**8**	**10**	**11**	**12**	**13**	**14**	**Average**	**St. Dev**	**N**	**St. Error**
6 PM	1	0	0	0	1	2	0	1	0	0	0	1	0	0.46	0.66	13.00	0.18
7 PM	0	1	1	0	0	0	0	0	2	0	2	3	0	0.69	1.03	13.00	0.29
8 PM	0	1	0	0	1	0	0	0	0	0	0	0	0	0.15	0.38	13.00	0.10
9 PM	0	0	0	0	0	0	0	0	0	0	0	0	0	0.00	0.00	13.00	0.00
10 PM	0	0	0	0	0	0	0	0	0	0	0	0	0	0.00	0.00	13.00	0.00
11 PM	0	0	0	0	0	0	0	0	3	0	0	0	0	0.23	0.83	13.00	0.23
12 AM	0	0	0	0	0	0	0	0	0	0	0	0	0	0.00	0.00	13.00	0.00
1 AM	0	0	0	0	0	0	0	0	0	0	0	0	0	0.00	0.00	13.00	0.00
2 AM	0	0	0	0	0	0	0	0	1	0	0	0	0	0.08	0.28	13.00	0.08
3 AM	0	0	0	0	0	0	0	0	0	0	0	0	0	0.00	0.00	13.00	0.00
4 AM	0	0	0	0	0	0	0	0	0	0	0	0	0	0.00	0.00	13.00	0.00
5 AM	0	0	2	3	0	0	2	0	0	1	2	0	0	0.77	1.09	13.00	0.30
6 AM	1	2	3	2	2	0	2	4	6	6	1	4	2	2.69	1.84	13.00	0.51
7 AM	0	2	4	1	0	4	4	2	3	3	1	5	3	2.46	1.61	13.00	0.45
8 AM	1	1	1	2	1	1	4	4	4	2	3	2	2	2.15	1.21	13.00	0.34
9 AM	0	2	1	1	0	0	0	1	2	1	0	2	1	0.85	0.80	13.00	0.22
10 AM	1	1	0	0	1	1	1	1	4	1	0	1	0	0.92	1.04	13.00	0.29

Cat

	Period																
**Hour**	**1**	**2**	**3**	**4**	**5**	**6**	**7**	**8**	**10**	**11**	**12**	**13**	**14**	**Average**	**St. Dev**	**N**	**St. Error**
6 PM	0	0	0	1	0	0	0	0	0	0	0	0	0	0.08	0.28	13.00	0.08
7 PM	0	0	0	0	0	0	1	0	0	0	0	0	0	0.08	0.28	13.00	0.08
8 PM	0	0	0	0	0	1	1	0	0	0	0	0	0	0.15	0.38	13.00	0.10
9 PM	0	0	0	0	1	0	0	0	0	0	0	0	1	0.15	0.38	13.00	0.10
10 PM	0	0	1	0	0	0	0	0	0	1	0	0	0	0.15	0.38	13.00	0.10
11 PM	0	0	0	0	0	0	0	0	0	0	0	0	0	0.00	0.00	13.00	0.00

12 AM	0	1	0	0	0	0	0	0	0	0	0	0	0	0.08	0.28	13.00	0.08
1 AM	0	1	0	0	0	0	0	0	0	1	0	0	0	0.15	0.38	13.00	0.10
2 AM	0	0	0	0	0	0	0	0	0	0	0	0	0	0.00	0.00	13.00	0.00
3 AM	0	0	0	0	0	0	0	0	0	0	0	1	0	0.08	0.28	13.00	0.08
4 AM	0	0	0	0	0	0	0	0	0	0	0	0	1	0.08	0.28	13.00	0.08
5 AM	0	0	0	1	0	0	0	0	0	3	0	0	1	0.38	0.87	13.00	0.24
6 AM	0	0	0	0	0	0	0	0	0	0	0	0	0	0.00	0.00	13.00	0.00
7 AM	0	0	0	0	0	0	0	0	0	0	0	0	0	0.00	0.00	13.00	0.00
8 AM	0	0	0	0	0	0	0	0	0	0	0	0	0	0.00	0.00	13.00	0.00
9 AM	0	0	0	0	0	0	0	0	0	0	0	0	0	0.00	0.00	13.00	0.00
10 AM	0	0	0	0	0	0	0	0	0	0	0	0	0	0.00	0.00	13.00	0.00

Hyena

	**Period**																
**Hour**	**1**	**2**	**3**	**4**	**5**	**6**	**7**	**8**	**10**	**11**	**12**	**13**	**14**	**Average**	**St. Dev**	**N**	**St. Error**
6 PM	0	0	0	0	0	0	0	0	0	0	0	0	0	0.00	0.00	13.00	0.00
7 PM	0	0	0	0	0	0	0	0	0	0	0	0	0	0.00	0.00	13.00	0.00
8 PM	0	0	0	0	0	0	0	0	0	0	0	0	0	0.00	0.00	13.00	0.00
9 PM	0	0	0	0	0	0	0	0	0	0	1	0	0	0.08	0.28	13.00	0.08
10 PM	0	0	0	0	0	0	1	0	0	0	0	0	1	0.15	0.38	13.00	0.10
11 PM	0	0	0	0	1	0	0	0	9	3	0	2	1	1.23	2.52	13.00	0.70
12 AM	0	1	0	1	0	0	0	0	6	2	0	2	4	1.23	1.88	13.00	0.52
1 AM	1	2	7	0	0	4	2	0	3	6	4	3	5	2.85	2.30	13.00	0.64
2 AM	4	2	2	1	3	1	4	0	1	11	2	2	1	2.62	2.79	13.00	0.77
3 AM	0	2	4	0	3	1	2	0	0	2	1	2	1	1.38	1.26	13.00	0.35
4 AM	0	0	1	0	0	0	0	0	1	0	0	0	0	0.15	0.38	13.00	0.10
5 AM	0	0	0	0	0	0	0	0	0	0	0	0	0	0.00	0.00	13.00	0.00
6 AM	0	0	0	0	0	0	0	0	0	0	0	0	0	0.00	0.00	13.00	0.00
7 AM	0	0	0	0	0	0	0	0	0	0	0	0	0	0.00	0.00	13.00	0.00
8 AM	0	0	0	0	0	0	0	0	0	0	0	0	0	0.00	0.00	13.00	0.00
9 AM	0	0	0	0	0	0	0	0	0	0	0	0	0	0.00	0.00	13.00	0.00
10 AM	0	0	0	0	0	0	0	0	0	0	0	0	0	0.00	0.00	13.00	0.00

Mongoose

Hour	Period 1	2	3	4	5	6	7	8	10	11	12	13	14	Average	St. Dev	N	St. Error
6 PM	0	0	0	0	0	0	0	0	0	0	0	0	0	0.00	0.00	13.00	0.00
7 PM	0	0	0	0	0	0	0	0	0	0	0	0	0	0.00	0.00	13.00	0.00
8 PM	0	0	0	0	0	0	0	0	0	0	0	0	0	0.00	0.00	13.00	0.00
9 PM	0	0	0	0	0	0	0	0	0	0	0	0	0	0.00	0.00	13.00	0.00
10 PM	1	0	0	0	0	0	0	0	0	0	0	0	0	0.08	0.28	13.00	0.08
11 PM	0	0	0	0	0	0	0	0	0	0	0	0	0	0.00	0.00	13.00	0.00
12 AM	0	0	0	0	0	0	1	0	1	0	0	0	0	0.15	0.38	13.00	0.10
1 AM	0	0	0	0	0	0	1	0	0	0	0	0	0	0.08	0.28	13.00	0.08
2 AM	1	0	1	0	0	0	0	1	0	0	0	0	0	0.23	0.44	13.00	0.12
3 AM	0	0	0	0	0	0	0	0	2	1	0	0	1	0.31	0.63	13.00	0.17
4 AM	0	0	0	0	0	0	1	0	0	1	1	1	0	0.31	0.48	13.00	0.13
5 AM	1	0	0	0	0	0	0	0	0	0	0	0	0	0.08	0.28	13.00	0.08
6 AM	0	0	0	0	0	0	0	0	0	0	0	0	0	0.00	0.00	13.00	0.00
7 AM	0	0	0	0	0	0	0	0	0	0	0	0	0	0.00	0.00	13.00	0.00
8 AM	0	0	0	0	0	0	0	0	0	0	0	0	0	0.00	0.00	13.00	0.00
9 AM	0	0	0	0	0	0	0	0	0	0	0	0	0	0.00	0.00	13.00	0.00
10 AM	0	0	0	0	0	0	0	0	0	0	0	0	0	0.00	0.00	13.00	0.00

Wolf

Hour	Period 1	2	3	4	5	6	7	8	10	11	12	13	14	Average	St. Dev	N	St. Error
6 PM	0	0	0	0	0	0	0	0	0	0	0	0	0	0.00	0.00	13.00	0.00
7 PM	0	0	0	0	0	0	0	0	0	0	0	0	0	0.00	0.00	13.00	0.00
8 PM	0	0	0	0	0	0	0	0	0	0	0	0	1	0.08	0.28	13.00	0.08
9 PM	0	0	0	0	0	0	0	0	0	0	0	0	0	0.00	0.00	13.00	0.00
10 PM	0	0	0	0	0	0	0	0	0	0	0	0	0	0.00	0.00	13.00	0.00
11 PM	0	0	0	0	0	0	0	0	0	0	0	0	0	0.00	0.00	13.00	0.00
12 AM	0	0	0	0	0	0	0	0	0	0	0	0	0	0.00	0.00	13.00	0.00
1 AM	0	0	0	0	0	0	0	0	0	0	0	0	0	0.00	0.00	13.00	0.00

2 AM	1	0	0	0	0	0	0	0	3	1	0	0	0	0.38	0.87	13.00	0.24
3 AM	0	0	0	0	0	0	0	0	2	0	0	1	1	0.31	0.63	13.00	0.17
4 AM	0	0	0	0	0	0	0	0	0	1	0	1	1	0.23	0.44	13.00	0.12
5 AM	0	0	0	0	0	0	0	0	0	0	0	1	0	0.08	0.28	13.00	0.08
6 AM	0	0	0	0	0	0	0	0	0	0	0	0	0	0.00	0.00	13.00	0.00
7 AM	0	0	0	0	0	0	0	0	0	0	0	0	0	0.00	0.00	13.00	0.00
8 AM	0	0	0	0	0	0	0	0	0	0	0	0	0	0.00	0.00	13.00	0.00
9 AM	0	0	0	0	0	0	0	0	0	0	0	0	0	0.00	0.00	13.00	0.00
10 AM	0	0	0	0	0	0	0	0	0	0	0	0	0	0.00	0.00	13.00	0.00

* Period # 9 excluded due to interference

Figure S1. Temporal trends in species composition and abundance at slaughter plants in Addis Ababa with standard error bars

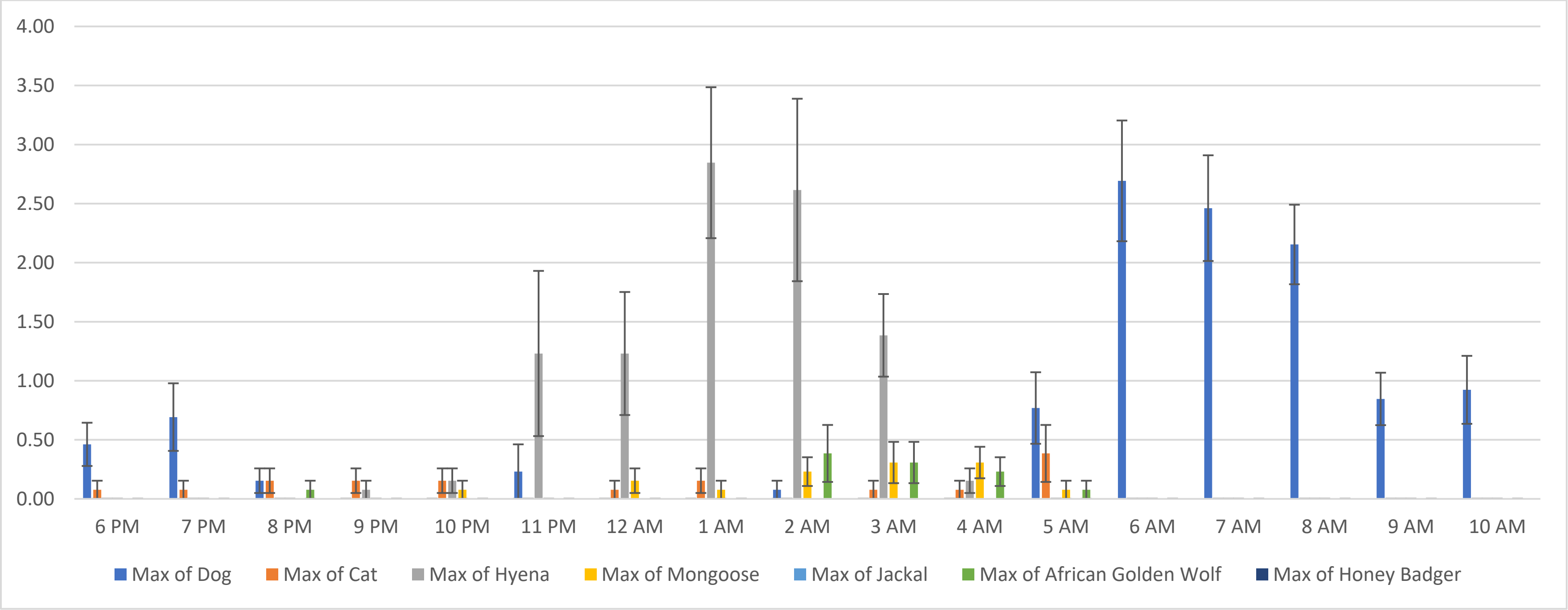

Table S4. Temporal trends in species composition and abundance at slaughter plants in Goba

Dog

	Period							
**Hour**	**1**	**2**	**3**	**4**	**Average**	**St. Dev**	**N**	**St. Error**
6 PM	2	1	2	4	2.25	1.26	4.00	0.63
7 PM	2	1	6	3	3.00	2.16	4.00	1.08
8 PM	1	2	4	1	2.00	1.41	4.00	0.71
9 PM	0	1	3	1	1.25	1.26	4.00	0.63
10 PM	1	1	3	1	1.50	1.00	4.00	0.50
11 PM	0	1	3	0	1.00	1.41	4.00	0.71
12 AM	0	0	2	1	0.75	0.96	4.00	0.48
1 AM	0	1	0	1	0.50	0.58	4.00	0.29
2 AM	0	0	2	1	0.75	0.96	4.00	0.48
3 AM	1	1	2	0	1.00	0.82	4.00	0.41
4 AM	1	1	1	1	1.00	0.00	4.00	0.00
5 AM	1	2	2	4	2.25	1.26	4.00	0.63
6 AM	12	10	33	8	15.75	11.62	4.00	5.81
7 AM	15	9	23		15.67	7.02	3.00	4.06
8 AM	11	6	14		10.33	4.04	3.00	2.33
9 AM	21	7	2		10.00	9.85	3.00	5.69
10 AM	23	11	15		16.33	6.11	3.00	3.53

Cat

	Period							
**Hour**	**1**	**2**	**3**	**4**	**Average**	**St. Dev**	**N**	**St. Error**
6 PM	0	0	0	0	0.00	0.00	4.00	0.00
7 PM	0	0	0	0	0.00	0.00	4.00	0.00
8 PM	0	0	0	0	0.00	0.00	4.00	0.00
9 PM	0	0	0	0	0.00	0.00	4.00	0.00
10 PM	0	0	0	0	0.00	0.00	4.00	0.00
11 PM	0	0	0	0	0.00	0.00	4.00	0.00

12 AM	1	0	0	0	0.25	0.50	4.00	0.25
1 AM	0	0	0	0	0.00	0.00	4.00	0.00
2 AM	0	0	0	0	0.00	0.00	4.00	0.00
3 AM	1	0	0	0	0.25	0.50	4.00	0.25
4 AM	0	0	1	1	0.50	0.58	4.00	0.29
5 AM	0	1	0	0	0.25	0.50	4.00	0.25
6 AM	0	0	0	0	0.00	0.00	4.00	0.00
7 AM	0	0	0		0.00	0.00	3.00	0.00
8 AM	0	0	0		0.00	0.00	3.00	0.00
9 AM	0	0	0		0.00	0.00	3.00	0.00
10 AM	0	0	0		0.00	0.00	3.00	0.00

Mongoose

	**Period**							
**Hour**	**1**	**2**	**3**	**4**	**Average**	**St. Dev**	**N**	**St. Error**
6 PM	0	0	0	0	0.00	0.00	4.00	0.00
7 PM	0	0	0	0	0.00	0.00	4.00	0.00
8 PM	0	0	1	0	0.25	0.50	4.00	0.25
9 PM	0	0	0	0	0.00	0.00	4.00	0.00
10 PM	0	0	0	0	0.00	0.00	4.00	0.00
11 PM	0	0	0	0	0.00	0.00	4.00	0.00
12 AM	0	0	0	0	0.00	0.00	4.00	0.00
1 AM	0	0	0	0	0.00	0.00	4.00	0.00
2 AM	0	0	0	0	0.00	0.00	4.00	0.00
3 AM	1	0	0	0	0.25	0.50	4.00	0.25
4 AM	0	0	0	0	0.00	0.00	4.00	0.00
5 AM	0	0	0	0	0.00	0.00	4.00	0.00
6 AM	0	0	0	0	0.00	0.00	4.00	0.00
7 AM	0	0	0		0.00	0.00	3.00	0.00
8 AM	0	0	0		0.00	0.00	3.00	0.00
9 AM	0	0	0		0.00	0.00	3.00	0.00
10 AM	0	0	0		0.00	0.00	3.00	0.00

Figure S2. Temporal trends in species composition and abundance at slaughter plants in Goba with standard error bars

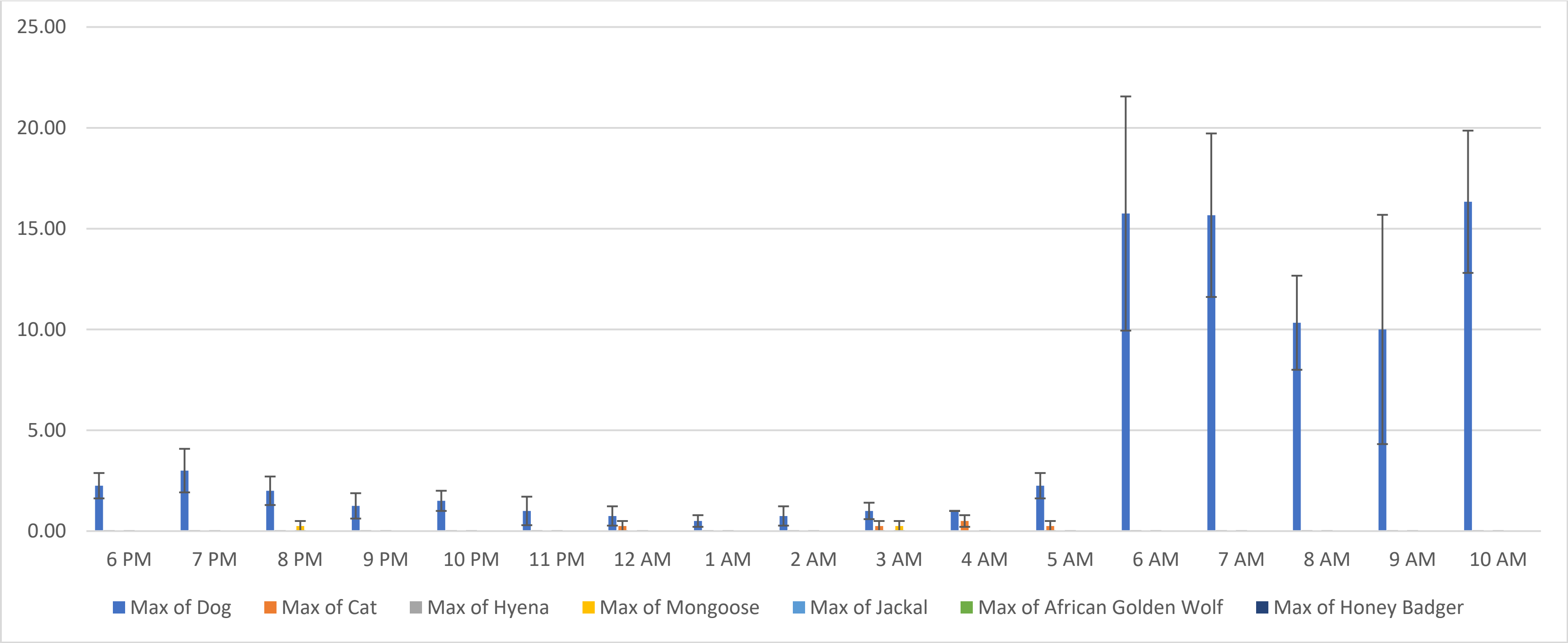

Table S5. Temporal trends in species composition and abundance at slaughter plants in Awash

			Dog						
	**Period**								
**Hour**	**1**	**2**	**3**	**4**	**5**	**Average**	**St. Dev**	**N**	**St. Error**
6 PM	1	1	1	1	1	1.00	0.00	5.00	0.00
7 PM	1	0	2	2	1	1.20	0.84	5.00	0.37
8 PM	1	0	2	0	1	0.80	0.84	5.00	0.37
9 PM	0	0	0	3	0	0.60	1.34	5.00	0.60
10 PM	0	0	0	0	0	0.00	0.00	5.00	0.00

11 PM	0	0	0	0	0	0.00	0.00	5.00	0.00
12 AM	0	0	0	0	0	0.00	0.00	5.00	0.00
1 AM	0	0	0	1	0	0.20	0.45	5.00	0.20
2 AM	0	1	0	0	0	0.20	0.45	5.00	0.20
3 AM	0	0	1	0	0	0.20	0.45	5.00	0.20
4 AM	1	0	1	0	0	0.40	0.55	5.00	0.24
5 AM	1	0	1	1	0	0.60	0.55	5.00	0.24
6 AM	1	2	1	2	1	1.40	0.55	5.00	0.24
7 AM	1	3	2	0		1.50	1.29	4.00	0.65
8 AM	1	1	0	1		0.75	0.50	4.00	0.25
9 AM	1	0	0	0		0.25	0.50	4.00	0.25
10 AM	0	0	0	0		0.00	0.00	4.00	0.00

Cat

	**Period**								
**Hour**	**1**	**2**	**3**	**4**	**5**	**Average**	**St. Dev**	**N**	**St. Error**
6 PM	0	1	0	1	0	0.40	0.55	5.00	0.24
7 PM	2	2	3	4	1	2.40	1.14	5.00	0.51
8 PM	1	3	2	4	2	2.40	1.14	5.00	0.51
9 PM	2	2	2	2	1	1.80	0.45	5.00	0.20
10 PM	2	1	1	2	2	1.60	0.55	5.00	0.24
11 PM	2	1	1	1	2	1.40	0.55	5.00	0.24
12 AM	1	0	0	1	1	0.60	0.55	5.00	0.24
1 AM	0	1	1	1	1	0.80	0.45	5.00	0.20
2 AM	0	3	1	1	1	1.20	1.10	5.00	0.49
3 AM	1	3	1	0	0	1.00	1.22	5.00	0.55
4 AM	1	2	1	0	1	1.00	0.71	5.00	0.32
5 AM	0	1	1	0	0	0.40	0.55	5.00	0.24
6 AM	0	0	0	0	0	0.00	0.00	5.00	0.00
7 AM	0	0	0	0		0.00	0.00	4.00	0.00
8 AM	0	0	0	0		0.00	0.00	4.00	0.00
9 AM	0	0	0	0		0.00	0.00	4.00	0.00

10 AM	0	0	0	0		0.00	0.00	4.00	0.00

Hyena

	Period								
**Hour**	**1**	**2**	**3**	**4**	**5**	**Average**	**St. Dev**	**N**	**St. Error**
6 PM	0	0	0	0	0	0.00	0.00	5.00	0.00
7 PM	0	0	0	0	0	0.00	0.00	5.00	0.00
8 PM	0	0	0	0	1	0.20	0.45	5.00	0.20
9 PM	0	0	1	1	1	0.60	0.55	5.00	0.24
10 PM	2	1	1	2	1	1.40	0.55	5.00	0.24
11 PM	1	1	0	2	0	0.80	0.84	5.00	0.37
12 AM	2	1	0	0	1	0.80	0.84	5.00	0.37
1 AM	1	1	1	0	1	0.80	0.45	5.00	0.20
2 AM	1	0	1	1	1	0.80	0.45	5.00	0.20
3 AM	1	0	0	1	1	0.60	0.55	5.00	0.24
4 AM	0	0	0	1	1	0.40	0.55	5.00	0.24
5 AM	0	0	0	0	0	0.00	0.00	5.00	0.00
6 AM	0	0	0	0	0	0.00	0.00	5.00	0.00
7 AM	0	0	0	0		0.00	0.00	4.00	0.00
8 AM	0	0	0	0		0.00	0.00	4.00	0.00
9 AM	0	0	0	0		0.00	0.00	4.00	0.00
10 AM	0	0	0	0		0.00	0.00	4.00	0.00

Mongoose

	Period								
**Hour**	**1**	**2**	**3**	**4**	**5**	**Average**	**St. Dev**	**N**	**St. Error**
6 PM	0	0	0	0	0	0.00	0.00	5.00	0.00
7 PM	0	0	0	0	0	0.00	0.00	5.00	0.00
8 PM	0	0	0	0	0	0.00	0.00	5.00	0.00
9 PM	0	0	0	0	0	0.00	0.00	5.00	0.00
10 PM	0	1	0	0	0	0.20	0.45	5.00	0.20
11 PM	0	0	0	0	0	0.00	0.00	5.00	0.00
12 AM	0	1	0	0	0	0.20	0.45	5.00	0.20

1 AM	0	0	0	0	1	0.20	0.45	5.00	0.20
2 AM	0	0	1	0	1	0.40	0.55	5.00	0.24
3 AM	0	0	1	0	0	0.20	0.45	5.00	0.20
4 AM	0	1	0	0	0	0.20	0.45	5.00	0.20
5 AM	0	0	0	0	0	0.00	0.00	5.00	0.00
6 AM	0	0	0	0	0	0.00	0.00	5.00	0.00
7 AM	0	0	0	0		0.00	0.00	4.00	0.00
8 AM	0	0	0	0		0.00	0.00	4.00	0.00
9 AM	0	0	0	0		0.00	0.00	4.00	0.00
10 AM	0	0	0	0		0.00	0.00	4.00	0.00

Honey Badger

	**Period**								
**Hour**	**1**	**2**	**3**	**4**	**5**	**Average**	**St. Dev**	**N**	**St. Error**
6 PM	0	0	0	0	0	0.00	0.00	5.00	0.00
7 PM	0	0	0	0	0	0.00	0.00	5.00	0.00
8 PM	1	0	0	0	0	0.20	0.45	5.00	0.20
9 PM	2	0	0	0	0	0.40	0.89	5.00	0.40
10 PM	0	0	0	0	0	0.00	0.00	5.00	0.00
11 PM	0	0	0	0	0	0.00	0.00	5.00	0.00
12 AM	2	0	0	0	0	0.40	0.89	5.00	0.40
1 AM	0	0	0	0	0	0.00	0.00	5.00	0.00
2 AM	0	0	0	0	0	0.00	0.00	5.00	0.00
3 AM	0	0	0	0	0	0.00	0.00	5.00	0.00
4 AM	0	0	0	0	0	0.00	0.00	5.00	0.00
5 AM	0	0	0	0	0	0.00	0.00	5.00	0.00
6 AM	0	0	0	0	0	0.00	0.00	5.00	0.00
7 AM	0	0	0	0		0.00	0.00	4.00	0.00
8 AM	0	0	0	0		0.00	0.00	4.00	0.00
9 AM	0	0	0	0		0.00	0.00	4.00	0.00
10 AM	0	0	0	0		0.00	0.00	4.00	0.00

Figure S3. Temporal trends in species composition and abundance at slaughter plants in Awash with standard error bars

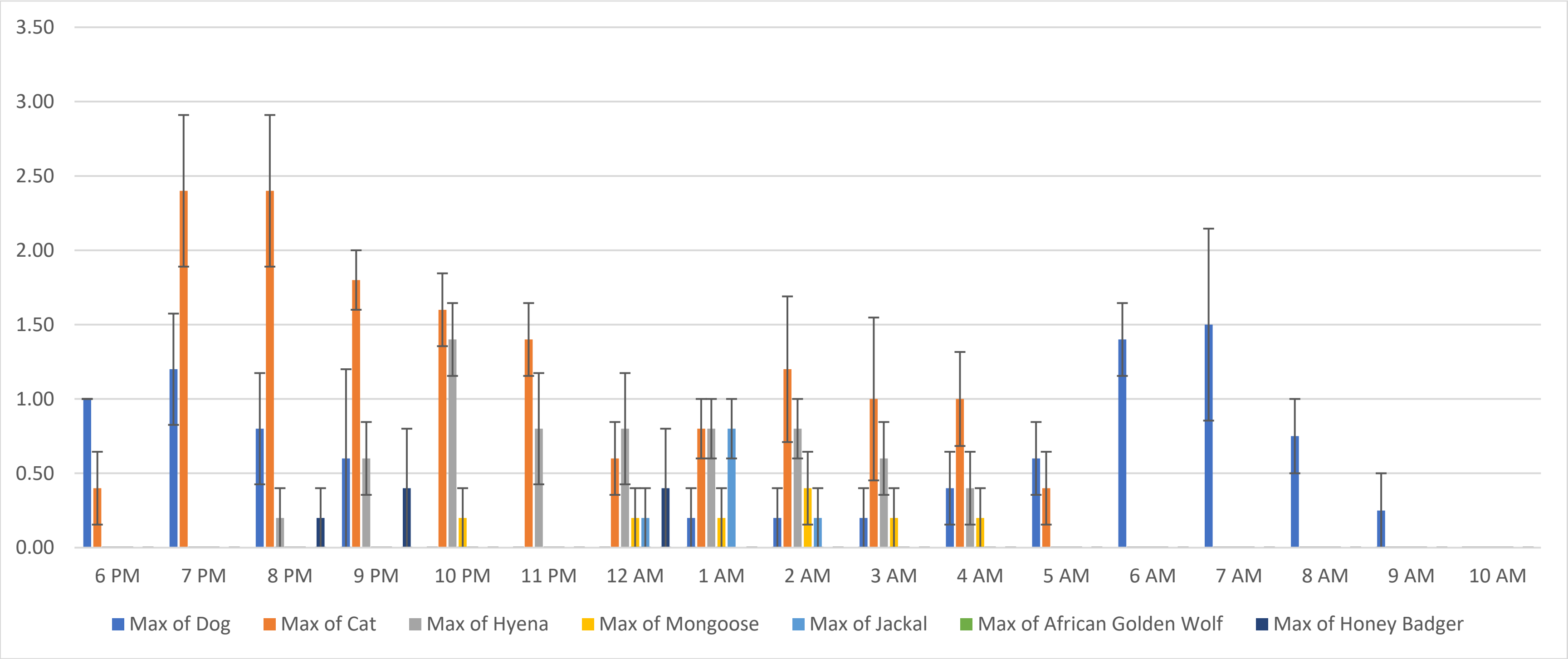

Table S6. Temporal trends in species composition and abundance at slaughter plants in Hawassa

	Dog						
	Period						
**Hour**	**1**	**2**	**3**	**Average**	**St. Dev**	**N**	**St. Error**
6 PM	0	0	0	0.00	0.00	3.00	0.00
7 PM	0	0	0	0.00	0.00	3.00	0.00
8 PM	0	0	0	0.00	0.00	3.00	0.00
9 PM	0	0	0	0.00	0.00	3.00	0.00
10 PM	0	0	0	0.00	0.00	3.00	0.00
11 PM	0	0	0	0.00	0.00	3.00	0.00

12 AM	0	0	0	0.00	0.00	3.00	0.00
1 AM	0	0	0	0.00	0.00	3.00	0.00
2 AM	0	0	0	0.00	0.00	3.00	0.00
3 AM	0	0	0	0.00	0.00	3.00	0.00
4 AM	0	0	0	0.00	0.00	3.00	0.00
5 AM	0	1	2	1.00	1.00	3.00	0.58
6 AM	2	3	4	3.00	1.00	3.00	0.58
7 AM	0	3	2	1.67	1.53	3.00	0.88
8 AM	0	0	0	0.00	0.00	3.00	0.00
9 AM	0	0	1	0.33	0.58	3.00	0.33
10 AM	0	0	0	0.00	0.00	3.00	0.00

Cat

	**Period**						
**Hour**	**1**	**2**	**3**	**Average**	**St. Dev**	**N**	**St. Error**
6 PM	0	0	0	0.00	0.00	3.00	0.00
7 PM	2	0	0	0.67	1.15	3.00	0.67
8 PM	2	0	0	0.67	1.15	3.00	0.67
9 PM	0	1	1	0.67	0.58	3.00	0.33
10 PM	0	2	0	0.67	1.15	3.00	0.67
11 PM	0	2	1	1.00	1.00	3.00	0.58
12 AM	1	2	0	1.00	1.00	3.00	0.58
1 AM	1	3	1	1.67	1.15	3.00	0.67
2 AM	3	4	3	3.33	0.58	3.00	0.33
3 AM	3	3	3	3.00	0.00	3.00	0.00
4 AM	0	1	2	1.00	1.00	3.00	0.58
5 AM	0	0	2	0.67	1.15	3.00	0.67
6 AM	0	0	0	0.00	0.00	3.00	0.00
7 AM	0	0	0	0.00	0.00	3.00	0.00
8 AM	0	0	0	0.00	0.00	3.00	0.00
9 AM	0	0	0	0.00	0.00	3.00	0.00
10 AM	0	0	0	0.00	0.00	3.00	0.00

Hyena

**Hour**	**Period** 1	**2**	**3**	**Average**	**St. Dev**	**N**	**St. Error**
6 PM	0	0	0	0.00	0.00	3.00	0.00
7 PM	0	0	0	0.00	0.00	3.00	0.00
8 PM	1	0	0	0.33	0.58	3.00	0.33
9 PM	0	0	0	0.00	0.00	3.00	0.00
10 PM	0	0	0	0.00	0.00	3.00	0.00
11 PM	3	0	1	1.33	1.53	3.00	0.88
12 AM	2	0	3	1.67	1.53	3.00	0.88
1 AM	4	3	2	3.00	1.00	3.00	0.58
2 AM	10	5	3	6.00	3.61	3.00	2.08
3 AM	6	5	4	5.00	1.00	3.00	0.58
4 AM	10	2	2	4.67	4.62	3.00	2.67
5 AM	5	3	2	3.33	1.53	3.00	0.88
6 AM	0	0	0	0.00	0.00	3.00	0.00
7 AM	0	0	0	0.00	0.00	3.00	0.00
8 AM	0	0	0	0.00	0.00	3.00	0.00
9 AM	0	0	0	0.00	0.00	3.00	0.00
10 AM	0	0	0	0.00	0.00	3.00	0.00

Figure S4. Temporal trends in species composition and abundance at slaughter plants in Hawassa with standard error bars

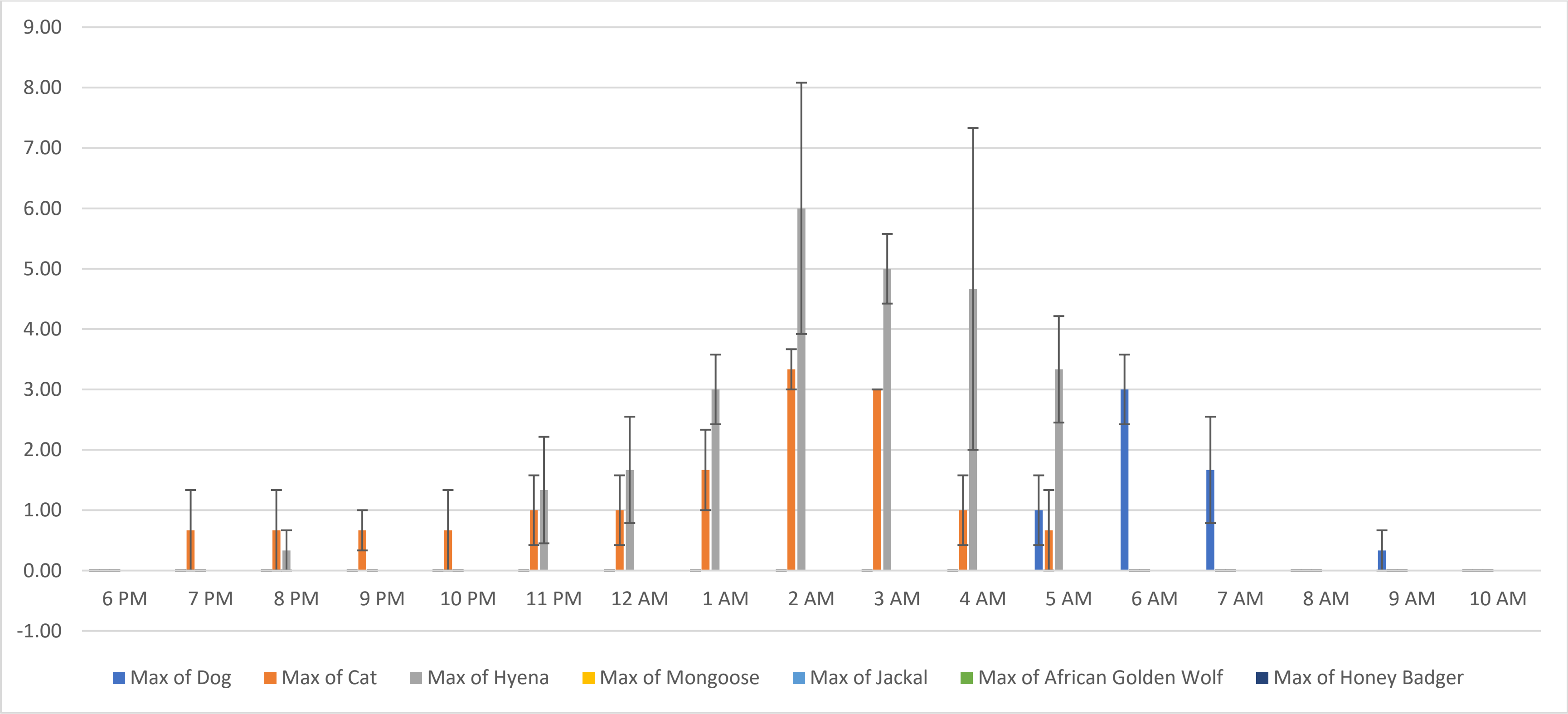

Table S7. Species observed during same hour but never observed together

**Species 1**	**Specie 2**	**Sites**
Jackal	Cat	Awash
Dog	Mongoose	Addis Ababa, Goba, Awash
Dog	Wolf	Addis Ababa
Hyena	Wolf	Addis Ababa
Cat	Wolf	Addis Ababa
Badger	Cat	Awash
Badger	Jackal	Awash
Badger	Mongoose	Awash
Badger	Hyena	Awash
Badger	Dog	Awash
Dog	Hyena	Addis Ababa, Awash, Hawassa
Dog	Jackal	Awash
Mongoose	Jackal	Awash

Table S8. Intra-species contact rates at all *v* threshold values.

**Site**	**Dog**			**Cat**			**Hyena**			**Mongoose**			**Wolf**			**Badger**		
v	**0.063**	**0.126**	**0.25**	**0.063**	**0.126**	**0.25**	**0.063**	**0.126**	**0.25**	**0.063**	**0.126**	**0.25**	**0.063**	**0.126**	**0.25**	**0.063**	**0.126**	**0.25**
**Addis Ababa**	0.5	1	1.98	0.03	0.06	0.11	0.66	1.32	2.62	0.02	0.03	0.06	0.03	0.06	0.11	0	0	0
**Goba**	3.39	6.78	13.45	0	0	0	0	0	0	0	0	0	0	0	0	0	0	0
**Awash**	0.19	0.38	0.76	0.34	0.69	1.36	0.08	0.15	0.3	0	0	0	0	0	0	0.04	0.08	0.15
**Hawassa**	0.38	0.76	1.52	0.44	0.89	1.77	1.02	2.04	4.04	0	0	0	0	0	0	0	0	0

Table S9. Contacts at Addis Ababa with potential transmission from the species listed horizontally to those listed vertically.

	**FROM**							
		Dog	*Cat*	*Hyena*	*Mongoose*	*Jackal*	*Wolf*	*Badger*
**TO**	*Dog*	2.62	0	0	0	0	0	0
	Cat	0	0.15	0.08	0	0	0	0
	Hyena	0	0.31	3.46	0.69	0	0	0
	Mongoose	0	0	0.31	0.08	0	0.08	0
	Jackal	0	0	0	0	0	0	0
	Wolf	0	0	0	0.08	0	0.15	0
	Badger	0	0	0	0	0	0	0

Table S10. Contacts at Goba with potential transmission from the species listed horizontally to those listed vertically.

	**FROM**							
		Dog	*Cat*	*Hyena*	*Mongoose*	*Jackal*	*Wolf*	*Badger*
**TO**	*Dog*	17.75	0	0	0	0	0	0
	Cat	0	0	0	0.25	0	0	0
	Hyena	0	0	0	0	0	0	0
	Mongoose	0	0.25	0	0	0	0	0
	Jackal	0	0	0	0	0	0	0
	Wolf	0	0	0	0	0	0	0
	Badger	0	0	0	0	0	0	0

Table S11. Contacts at Awash with potential transmission from the species listed horizontally to those listed vertically.

	**FROM**							
		Dog	*Cat*	*Hyena*	*Mongoose*	*Jackal*	*Wolf*	*Badger*
**TO**	*Dog*	1	1.4	0	0	0	0	0
	Cat	1.6	1.8	1	0.2	0	0	0
	Hyena	0	1	0.4	0	0.6	0	0
	Mongoose	0	0.2	0	0	0	0	0
	Jackal	0	0	0.6	0	0	0	0
	Wolf	0	0	0	0	0	0	0
	Badger	0	0	0	0	0	0	0.2

Table S12. Contacts at Awassa with potential transmission from the species listed horizontally to those listed vertically.

	**FROM**							
		Dog	*Cat*	*Hyena*	*Mongoose*	*Jackal*	*Wolf*	*Badger*
**TO**	*Dog*	2	0	0	0	0	0	0
	Cat	0	2.33	3	0	0	0	0
	Hyena	0	6.33	5.33	0	0	0	0
	Mongoose	0	0	0	0	0	0	0
	Jackal	0	0	0	0	0	0	0
	Wolf	0	0	0	0	0	0	0
	Badger	0	0	0	0	0	0	0

Table S13. Inter-species contact rates at all *v* threshold values.

Site	**Dog-Cat**			**Cat-Hyena**			**Jackal- Hyena**			**Hyena-Mongoose**			**Wolf-Mongoose**			**Mongoose-Cat**		
R0	***0.063***	***0.126***	***0.25***	***0.063***	***0.126***	***0.25***	***0.063***	***0.126***	***0.25***	***0.063***	***0.126***	***0.25***	***0.063***	***0.126***	***0.25***	***0.063***	***0.126***	***0.25***
Addis Ababa	0	0	0	0.69	1.38	2.74	0	0	0	0.69	1.37	2.73	0.05	0.10	0.19	0	0	0
Goba	0	0	0	0	0	0	0	0	0	0	0	0	0	0	0	0.05	0.1	0.19
Awash	0.66	1.32	2.61	0.49	0.99	*1.96*	0.16	0.32	0.63	0	0	0	0	0	0	0.35	0.7	1.38
Hawassa	0	0	0	1.84	3.68	*7.3*	0	0	0	0	0	0	0	0	0	0	0	0
